# Longitudinal Assessment of SARS-CoV-2-Specific T Cell Cytokine-Producing Responses for 1 Year Reveals Persistence of Multicytokine Proliferative Responses, with Greater Immunity Associated with Disease Severity

**DOI:** 10.1128/jvi.00509-22

**Published:** 2022-06-14

**Authors:** Jonah Lin, Ryan Law, Chapin S. Korosec, Christine Zhou, Wan Hon Koh, Mohammad Sajjad Ghaemi, Philip Samaan, Hsu Kiang Ooi, Vitaliy Matveev, FengYun Yue, Anne-Claude Gingras, Antonio Estacio, Megan Buchholz, Patti Lou Cheatley, Avid Mohammadi, Rupert Kaul, Katerina Pavinski, Samira Mubareka, Allison J. McGeer, Jerome A. Leis, Jane M. Heffernan, Mario Ostrowski

**Affiliations:** a Department of Immunology, University of Toronto, Toronto, Ontario, Canada; b Department of Medicine, University of Toronto, Toronto, Ontario, Canada; c Institute of Medical Science, University of Toronto, Toronto, Ontario, Canada; d Lunenfeld-Tanenbaum Research Institute at Mt. Sinai Hospital, Sinai Health System, Toronto, Ontario, Canada; e Keenan Research Centre for Biomedical Science of St. Michael’s Hospital, Unity Health, Toronto, Ontario, Canada; f Modelling Infection and Immunity Lab, Centre for Disease Modelling, Department of Mathematics and Statistics, York University, Toronto, Ontario, Canada; g Department of Laboratory Medicine, St. Michael’s Hospital, Unity Health, Toronto, Ontario, Canada; h Apheresis Unit, Kidney and Metabolism Program, St. Michael’s Hospital, Unity Health, Toronto, Ontario, Canada; i Department of Laboratory Medicine and Pathobiology, University of Toronto, Toronto, Ontario, Canada; j Digital Technologies Research Centre, National Research Council Canada, Toronto, Ontario, Canada; k Sunnybrook Health Sciences Centre and Research Institute, Toronto, Ontario, Canada; l Department of Medical Genetics, University of Toronto, Toronto, Ontario, Canada; University of North Carolina at Chapel Hill

**Keywords:** ELISpot assay, SARS-CoV-2, T cell immunity, cytokines, granzyme B, immune modeling

## Abstract

Cell-mediated immunity is critical for long-term protection against most viral infections, including coronaviruses. We studied 23 severe acute respiratory syndrome coronavirus 2 (SARS-CoV-2)-infected survivors over a 1-year post-symptom onset (PSO) interval by *ex vivo* cytokine enzyme-linked immunosorbent spot assay (ELISpot) assay. All subjects demonstrated SARS-CoV-2-specific gamma interferon (IFN-γ), interleukin 2 (IL-2), and granzyme B (GzmB) T cell responses at presentation, with greater frequencies in severe disease. Cytokines, mainly produced by CD4^+^ T cells, targeted all structural proteins (nucleocapsid, membrane, and spike) except envelope, with GzmB and IL-2 greater than IFN-γ. Mathematical modeling predicted that (i) cytokine responses peaked at 6 days for IFN-γ, 36 days for IL-2, and 7 days for GzmB, (ii) severe illness was associated with reduced IFN-γ and GzmB but increased IL-2 production rates, and (iii) males displayed greater production of IFN-γ, whereas females produced more GzmB. *Ex vivo* responses declined over time, with persistence of IL-2 in 86% and of IFN-γ and GzmB in 70% of subjects at a median of 336 days PSO. The average half-life of SARS-CoV-2-specific cytokine-producing cells was modeled to be 139 days (~4.6 months). Potent T cell proliferative responses persisted throughout observation, were CD4 dominant, and were capable of producing all 3 cytokines. Several immunodominant CD4 and CD8 epitopes identified in this study were shared by seasonal coronaviruses or SARS-CoV-1 in the nucleocapsid and membrane regions. Both SARS-CoV-2-specific CD4^+^ and CD8^+^ T cell clones were able to kill target cells, though CD8 tended to be more potent.

**IMPORTANCE** Our findings highlight the relative importance of SARS-CoV-2-specific GzmB-producing T cell responses in SARS-CoV-2 control and shared CD4 and CD8 immunodominant epitopes in seasonal coronaviruses or SARS-CoV-1, and they indicate robust persistence of T cell memory at least 1 year after infection. Our findings should inform future strategies to induce T cell vaccines against SARS-CoV-2 and other coronaviruses.

## INTRODUCTION

As of November 2021, the World Health Organization (WHO) had reported over 250 million confirmed cases and more than 5 million deaths due to COVID-19 (1). Severe acute respiratory syndrome coronavirus 2 (SARS-CoV-2) spike antibody-inducing vaccines have dramatically slowed the infection and death rate in the developed world; however, breakthrough infections due to delta and other variant viruses continue, and infection rates continue to rise in nonvaccinated regions (2–7).

Neutralizing antibodies against SARS-CoV-2 spike are a major effector in protection against infection and disease. However, some level of protection has been observed in vaccinated individuals prior to serum neutralizing antibody development, indicating that other arms of the immune system likely play a role (8). Also, individuals with recent other beta coronavirus infections who do not have cross-reactive antibodies appear to have more limited disease after SARS-CoV-2 infection (9), suggesting mediators of protection other than antibodies. T cells are generally important for viral clearance and disease protection, whereby CD4^+^ T cells can enhance antibody maturation and CD8^+^ T cell-mediated killing of infected cells. In regard to SARS-CoV-2 infection, a potent early T cell response correlates with disease outcome (10) and T cell responses are induced during convalescence (11–13), which have been shown to persist for up to 8 months (14).

A number of important questions remain regarding the role of T cells in COVID-19 immunity and disease. The roles of CD4^+^ versus CD8^+^ T cell memory in disease and protection are unclear. The relative importance of various T cell effector cytokines such as gamma interferon (IFN-γ), interleukin 2 (IL-2), and granzyme B (GzmB) is still poorly understood. It is suspected that mapping of CD4^+^ and CD8^+^ T cell epitopes and with attention to regions outside spike protein may inform the next generation of SARS-CoV-2 vaccines, given the emergence of antibody escape variants in vaccinated persons. It is still unclear how long T cell memory can persist in SARS-CoV-2 infection, given that reinfection is very common with coronaviruses in general (15, 16), albeit often with reduced severity. Such information should in the future reveal how T cells operate as correlates of protection against infection and disease.

In the current study, we prospectively monitored a cohort of SARS-CoV-2-infected individuals with various levels of disease outcome by using highly sensitive cytokine enzyme-linked immunosorbent spot (ELISpot) assays to follow SARS-CoV-2 antigen-specific cytokine responses averaging about 1 year of follow-up. We modeled the kinetics of the T cell cytokine response with time. In addition, we mapped a panel of epitopes to structural proteins that characterized dominant T cell responses in this cohort and show effector function and killing capabilities of both CD4^+^ and CD8^+^ T cell clones.

## RESULTS

### Clinical data of participants.

Clinical data of SARS-CoV-2-infected subjects can be found in Table 1. Twenty-three subjects were sampled at the time of presentation, during acute infection and/or shortly after convalescence, ranging between 7 and 160 days post-symptom onset (PSO). However, only 21 subjects were available for subsequent blood draws to understand long-term persistence of immune memory to SARS-CoV-2 (2 to 7 blood draws up to 398 days PSO). All subjects were infected with the ancestral circulating Wuhan strain. Disease severity ranged from mild illness (asymptomatic or symptomatic upper or lower respiratory tract symptoms but not requiring hospital admission, nonhospitalized, WHO classification mild [17]) to moderate illness (moderate disease, symptoms requiring hospital admission, unstable clinical status, partial O_2_ pressure (pO_2_) saturation of <94% on room air, radiologic evidence of pneumonia, WHO classification severe disease) to severe illness (intensive care unit [ICU] admission, WHO classification critical). Out of the 23 subjects studied, 9 were female (39%) and 14 were male (61%), with an average age of 50 years (range, 23 to 72 years); 14 had mild (61%), 6 had moderate (26%), and 3 had severe (13%) COVID-19 disease. Subjects with moderate and severe disease had median hospital stay durations of 4 and 14 days, respectively. Two subjects (OM8100 and OM8123) received one dose of the Pfizer BNT162b2 COVID-19 mRNA vaccine in between study visits. Another subject (OM8126) received two doses of the Pfizer vaccine in between study visits. Healthy control subjects included individuals who either had blood drawn pre-COVID (before January 2020) or were asymptomatic with no history of viral illness and had negative SARS-CoV-2 serology. Three individuals who were hospitalized were sampled intensively at 7- to 10-day intervals during and after their hospital admission for 6 weeks. The remainder of individuals were sampled during their convalescence after most symptoms had resolved.

**TABLE 1:** Clinical summary of SARS-CoV-2-infected subjects

Parameter	Value for patients with indicated level of disease			Total
	Asymptomatic/mild	Moderate	Severe	
No. of participants (%)	14 (61%)	6 (26%)	3 (13%)	23
Male/female (*n*)	7/7	4/2	3/0	14/9
Age (yr), mean ± SD	47 ± 14	59 ± 9	49 ± 6	50 ± 13
Days in hospital, median; range	None	4; 1–16	14; 11–15	8; 1–16
Sample collection range (first visit)	9–160 days PSO	7–78 days PSO	29–34 days PSO	Median: 36 days PSO
Sample collection range (last visit)	191–392 days PSO	169–360 days PSO	377–398 days PSO	Median: 336 days PSO

### Cytokine effector T cell responses at presentation.

**FIG 1:**
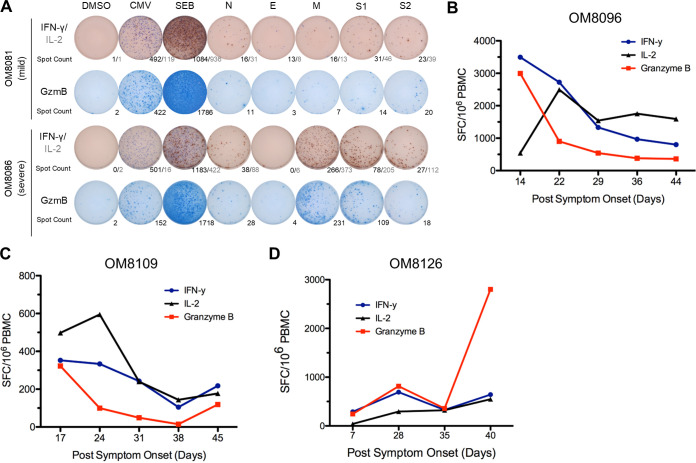
Stronger overall T cell cytokine ELISpot assay responses detected in patients with severe disease and overall ELISpot assay responses of acute patients in the first 6 weeks PSO. (A) Representative cytokine ELISpot assay responses of patients with mild (*n* = 13) and severe (*n* = 2) disease against SARS-CoV-2 structural proteins (N, E, M, S1, and S2), with DMSO as a negative control and cytomegalovirus (CMV) and SEB as positive controls. Numbers indicate the number of spot-forming cells for IFN-γ/IL-2 or GzmB. (B to D) Three patients with acute moderate COVID-19 infection were followed weekly shortly after symptom onset for up to 6 weeks PSO. Total additive response to the four SARS-CoV-2 structural proteins was measured by ELISpot assay for each cytokine.

Effector T cell responses that include IFN-γ (18), IL-2 (19), and GzmB (20) production are important in viral control of a Th1-mediated antiviral response. Cytokine ELISpot assay is a highly sensitive methodology to detect low-frequency viral antigen-specific responses in *ex vivo* peripheral blood mononuclear cells (PBMC) during or after virus infections (21). We measured SARS-CoV-2 antigen-specific IFN-γ, IL-2, and GzmB responses at presentation by cytokine ELISpot assay. We measured responses to the structural proteins of SARS-CoV-2, which included spike (S1 and S2), membrane (M), nucleocapsid (N), and envelope (E) (Fig. 1A). In addition, we included a peptide pool only spanning spike receptor binding domain and transmembrane domains (S-RBD and S-TM) in certain experiments to further evaluate T cell responses to this region. A representative example of cytokine ELISpot assays performed on *ex vivo* PBMC is shown in Fig. 1A.

### T cell immune responses during acute infection in hospitalized individuals with moderate illness.

We studied 3 individuals during the acute stage of SARS-CoV-2 infection, all who were hospitalized (moderate disease), over a 6-week period from days 7 to 44 PSO and assessed cytokine ELISpot responses over this period at about 7- to 10-day intervals. Total effector cytokine SARS-CoV-2-specific responses are depicted in Fig. 1B to D for each individual. Although all three individuals had moderate disease and were discharged from the hospital, we found considerable dynamic fluctuations in their early T cell cytokine responses to SARS-CoV-2 antigens. Cytokine antigen-specific responses were detectable at all time points. We tended to see an early peak of T cell cytokine responses in the first 22 to 28 days, followed by a leveling of the response afterwards.

### Memory SARS-CoV-2 T cell responses in convalescent patients.

**FIG 2:**
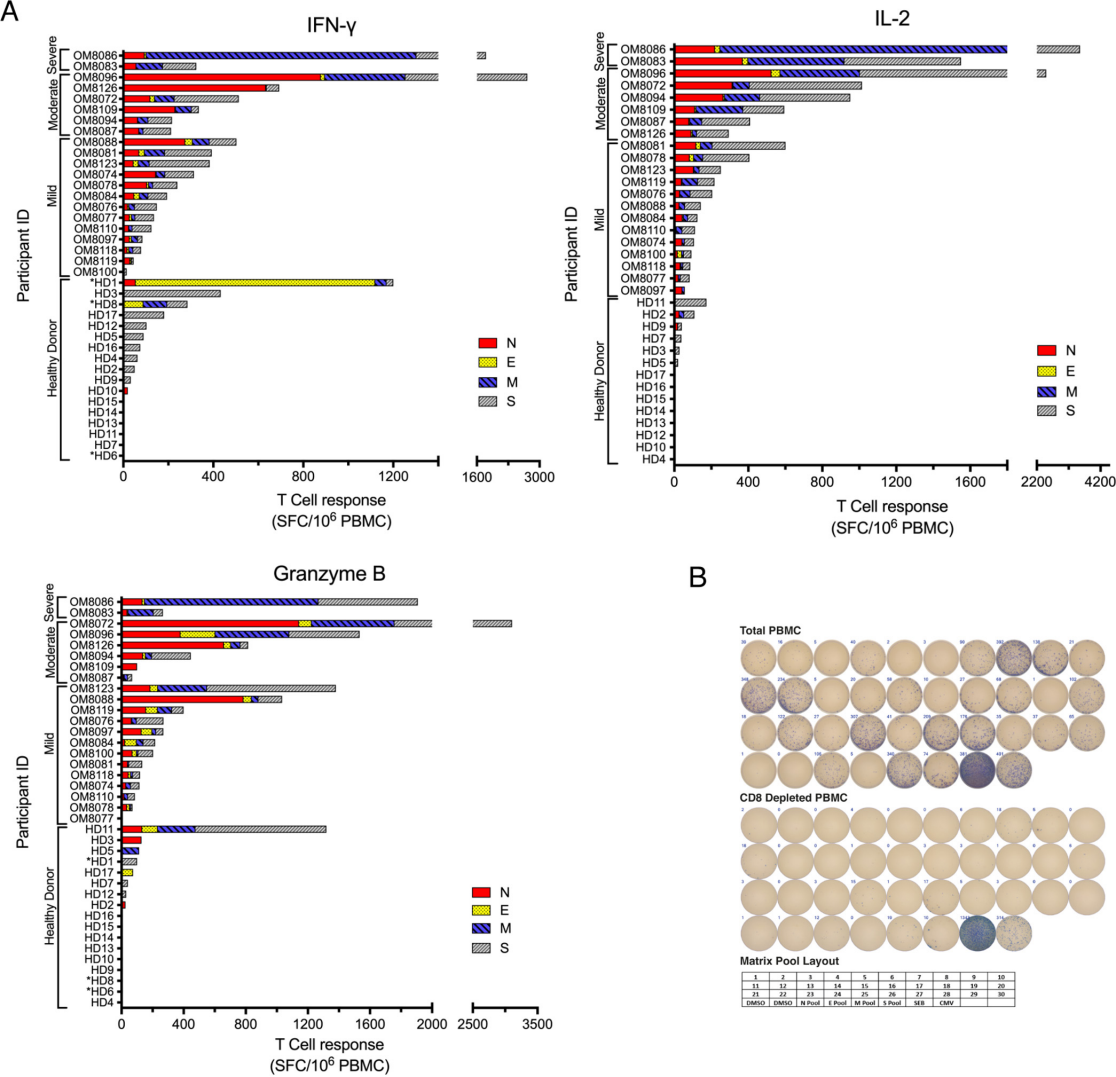
Summary of peak T cell responses during convalescence and in healthy donors (HD). (A) Overall peak ELISpot assay responses against SARS-CoV-2 structural proteins (N/E/M/S1/S2) in convalescent patients with mild, moderate, and severe disease as well as in nonsymptomatic healthy donors. Spot numbers were combined for each structural protein, and the peak total response within each patient’s time points was selected for IFN-γ, IL-2, and GzmB. Healthy donors indicated with an asterisk were assayed with spike peptide pool containing only the RBD and TM regions. HD1 to -11 were asymptomatic, seronegative, postpandemic donors with no recent respiratory illness; HD12 to -17 were healthy prepandemic donors. (B) Comparison of PBMC and CD4-depleted peptide matrix IFN-γ ELISpot assay responses. IFN-γ ELISpot assay responses of PBMC from a subject with severe disease (OM8086) and CD4-depleted PBMC against SARS-CoV-2 structural proteins (N/E/M/S-RBD+TM) via matrix and total peptide pools, and with complete DMSO as a negative control and CMV and SEB as positive controls. IFN-γ ELISpot assay responses were reduced significantly (>90%; 2,617 total spots in PBMC versus 190 in CD4-depleted PBMC) in the majority of the wells after CD4 depletion. The bottom shows the plate layout used for the peptide matrix IFN-γ ELISpot assay.

To examine the duration and the strength of the T cell responses against SARS-CoV-2 structural proteins (N, E, M, S1 [5′ region of spike with RBD], S2 [3′ region of spike after RBD]), 21 convalescent (most symptoms resolved) subjects (13 with mild, 6 with moderate, and 2 with severe disease) were examined longitudinally via *ex vivo* cytokine ELISpot assay, where the median time PSO of first blood draw was 38 days (range, 7 to 160 days PSO). For the majority of subjects, the first time point sampled was when we usually observed the most potent cytokine responses. A summary of all cytokine responses of all individuals during their maximal response during convalescence is depicted in Fig. 2A. As for the most frequent T cell targets of SARS-CoV-2 structural proteins, 7/21 (33%) subjects responded to N most frequently, followed by S2 with 6/21 (29%) subjects, S1 with 5/21 (24%) subjects, and M with 4/21 (19%) subjects. Minimal to no responses to E protein were observed. The greatest frequencies of cytokine-producing cells were found in those with moderate and severe disease rather than mild disease. Overall, GzmB- and IL-2-inducing cytokine responses from *ex vivo* T cells were greater than for IFN-γ. For the 21 subjects, the mean peak SARS-CoV-2 responses were as follows: for IL-2, 635 ± 198 spot-forming cells (SFC)/10^6^ PBMC; for GzmB, 597 ± 172 SFC/10^6^ PBMC; and for IFN-γ, 451 ± 140 SFC/10^6^ PBMC (IL-2 versus IFN-γ, *P* = 0.046; GzmB versus IFN- γ, *P* = 0.05; IL-2 versus GzmB, not significant [ns]; Wilcoxon rank sum test). Among the 21 participants, 11 exhibited GzmB responses as their strongest cytokine response, which were against mainly N and S1/S2. Seven participants possessed IL-2 responses as their strongest response, which were mainly against M and S1/S2. Only 3 subjects exhibited IFN-γ as their strongest responses, which targeted mainly N and S1/S2. Total *ex vivo* SARS-CoV-2-specific frequencies for subjects with severe/moderate disease versus those with mild disease were as follows: for IFN-γ, 852 versus 204 SFC/10^6^ PBMC (*P* = 0.02); for IL-2, 1,358 versus 191 SFC/10^6^ PBMC (*P* < 0.001); and for GzmB, 1,030 versus 331 SFC/10^6^ PBMC (*P* = 0.023), respectively, thus indicating that those with severe/moderate disease had higher frequencies of cytokine-producing SARS-CoV-2-specific T cells than those who suffered mild disease during convalescence. The majority of the cytokine response was contributed by CD4^+^ T cells, since CD4^+^ T cell depletion of PBMC resulted in reduction of all cytokine responses by 90% (range, 70 to 95% [Fig. 2B and data not shown]). In order to further understand the intensity of SARS-CoV-2-specific T cell responses in convalescent individuals, we also looked at uninfected individuals (pre-2020 and asymptomatic SARS-CoV-2-seronegative individuals) since preexisting immunity to seasonal alpha and beta coronaviruses may impart cross-reactive immune responses to SARS-CoV-2 antigens, as recently demonstrated (12, 22, 23). We found that at least 50% of uninfected and pre-COVID-19 individuals demonstrated cross-reactive immune responses to SARS-CoV-2 proteins (Fig. 2A), with 11/17 SARS-CoV-2-negative individuals having detectable IFN-γ responses, which were generally observed at lower frequencies than for convalescent individuals.

**FIG 3:**
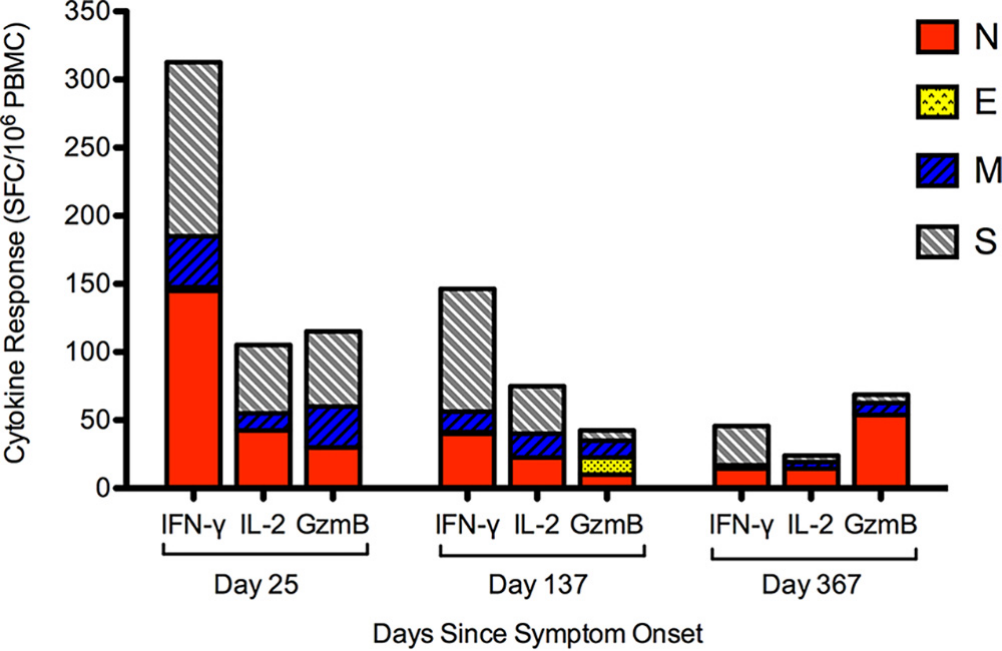
Representative longitudinal ELISpot assay data demonstrating decay of cytokine responses against SARS-CoV-2 structural proteins over time. Overall IFN-γ, IL-2, and GzmB ELISpot assay responses against SARS-CoV-2 structural proteins (N, E, M, S1, and S2) were measured over a period of 1 year PSO.

We followed *ex vivo* cytokine responses over a 1-year period (range, 169 to 398 days PSO). In general, we saw a decline in *ex vivo* SARS-CoV-2 responses to all antigens and cytokines. A representative example of one individual is shown in Fig. 3. To understand the decay of immune memory, the frequencies of low-level SARS-CoV-2 *ex vivo* responses that declined to below 50 SFC/10^6^ PBMC to all antigens combined (N, E, M, and S1 plus S2) were 33% for IFN-γ, 14% for IL-2, and 29% for GzmB at a median time point of 336 days PSO. Three individuals also received BNT162b2 vaccine during follow-up and showed variable ELISpot responses postvaccination: one subject (OM8100) showed increased IFN-γ/IL-2/GzmB responses to spike (S1 plus S2) but continued decay of N and M after vaccination, the second subject (OM8123) showed continued decay of responses after vaccination, and the third subject (OM8126) showed only increased IFN-γ responses only to S1 and no changes against other proteins (data not shown).

### Modeling of *ex vivo* SARS-CoV-2-specific immunity over time.

**FIG 4:**
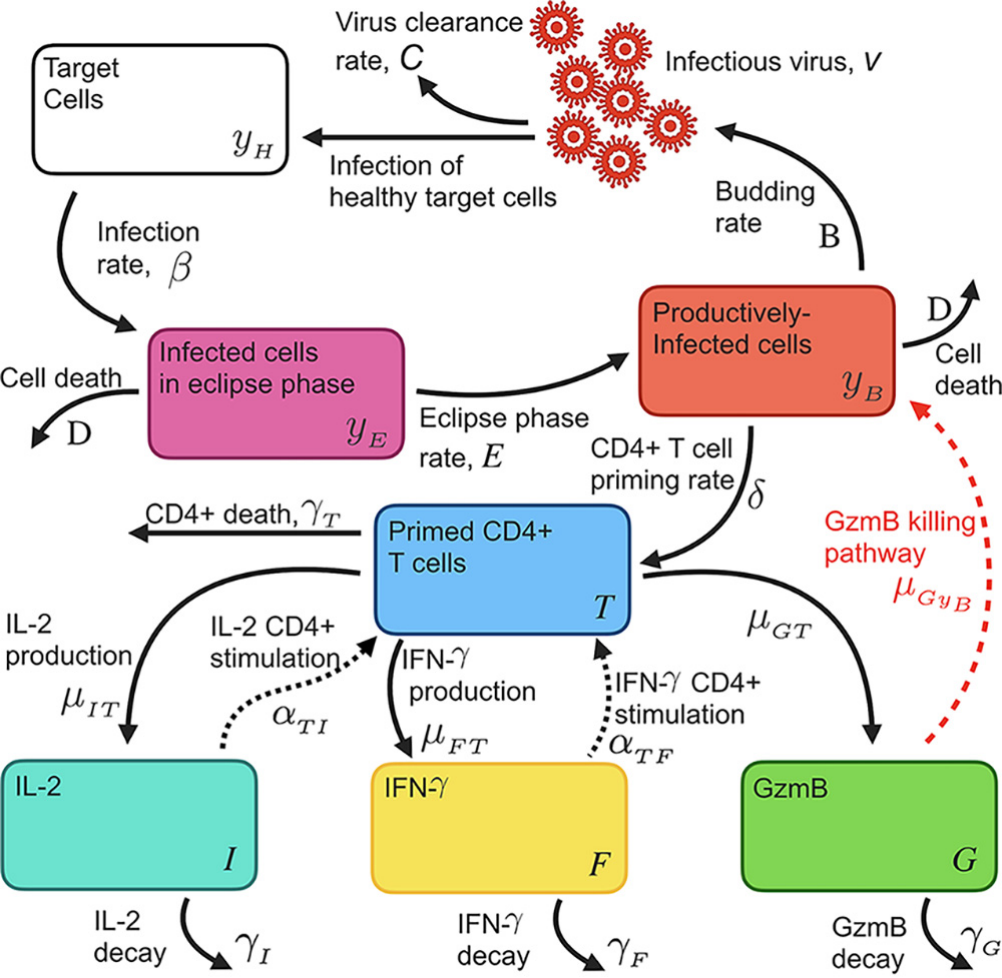
Schematic diagram of SARS-CoV-2 viral infection, CD4^+^ stimulation, and basic inflammatory response model (equations 1 to 8). Briefly, target cells (*y_H_*) are infected by virus (*v*) at rate β and then transition to an infected eclipse phase (*y_E_*). Cells in the eclipse phase turn over to productively infected cells (*y_B_*) at rate *E* and bud infectious virus at rate *B*. Infectious virus is cleared from the system at rate *C* and is, in turn, capable of infecting healthy target cells. CD4^+^ T cell priming occurs at rate δ and is proportional to the budding cell population (where δ ≪ 1). Primed CD4^+^ T cells (*T*) then produce cytokines IL-2 (*I*) and IFN-γ (*F*) at rates μ_IT_ and μ_FT_, respectively, and GzmB (G) at rate μ_GT_. We allow cytokines to influence the rate of CD4^+^ priming through terms α_TI_ and α_TF_ for IL-2 and IFN-γ, respectively. IL-2, IFN-γ, and GzmB degrade at rates γ_I_, γ_F_, and γ_G_, respectively.

**FIG 5:**
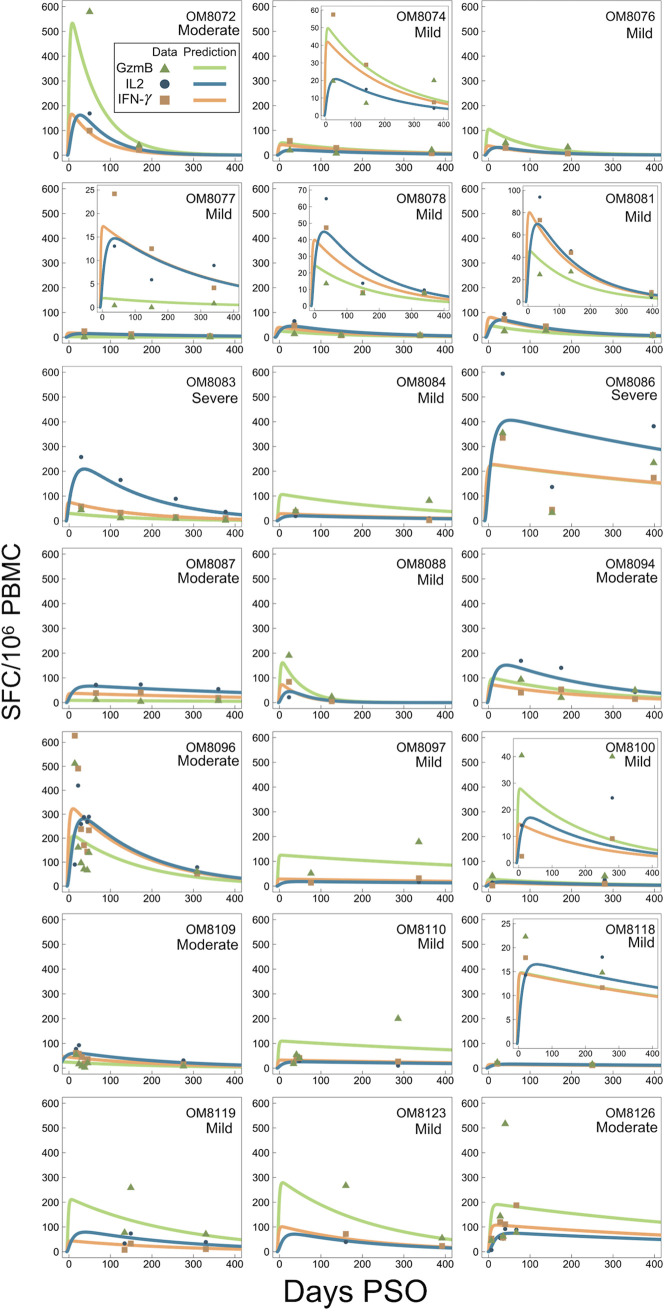
Individual fits as a function of days PSO to the kinetic model (equations 1 to 8). Solid lines are fits to the model in equations 1 to 8, where IL-2, IFN-γ, and GzmB are fit to equations 6, 7, and 8, respectively.

**FIG 6:**
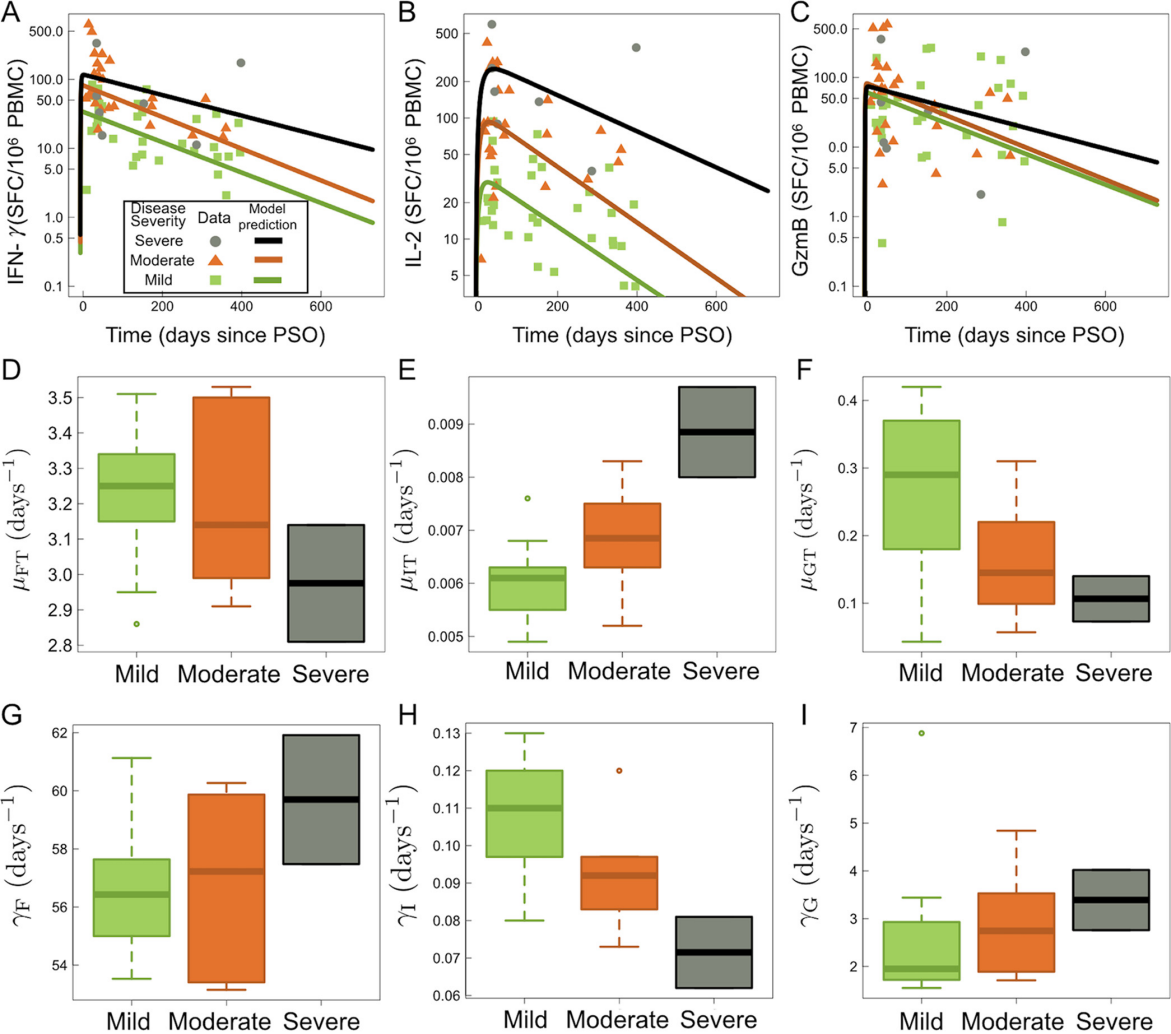
Kinetic model (equations 1 to 8) results sorted by severity. Clinical data in panels A to C are separated by case severity: severe, moderate, and mild. (A) IFN-γ data and fits to equation 7 as a function of days since PSO. (B) IL-2 data and fits to equation 6 as a function of days since PSO. (C) GzmB data and fits to equation 8 as a function of days since PSO. Panels D to I show boxplots of model fitted parameters for equations 6, 7, and 8 sorted by case severity. (D) IFN-γ stimulation rate by CD4^+^ T cells, μ_FT_, for mild, moderate, and severe disease. (E) IL-2 stimulation rate by CD4^+^ T cells, μ_ΙΤ_, for mild, moderate, and severe disease. (F) GzmB stimulation rate by CD4^+^ T cells, μ_GT_, for mild, moderate, and severe disease. (G) IFN-γ degradation rate, γ_F_, for mild, moderate, and severe disease. (H) IL-2 degradation rate, γ_I_, for mild, moderate, and severe disease. (I) GzmB degradation rate, γ_G_, for mild, moderate, and severe disease.

**FIG 7:**
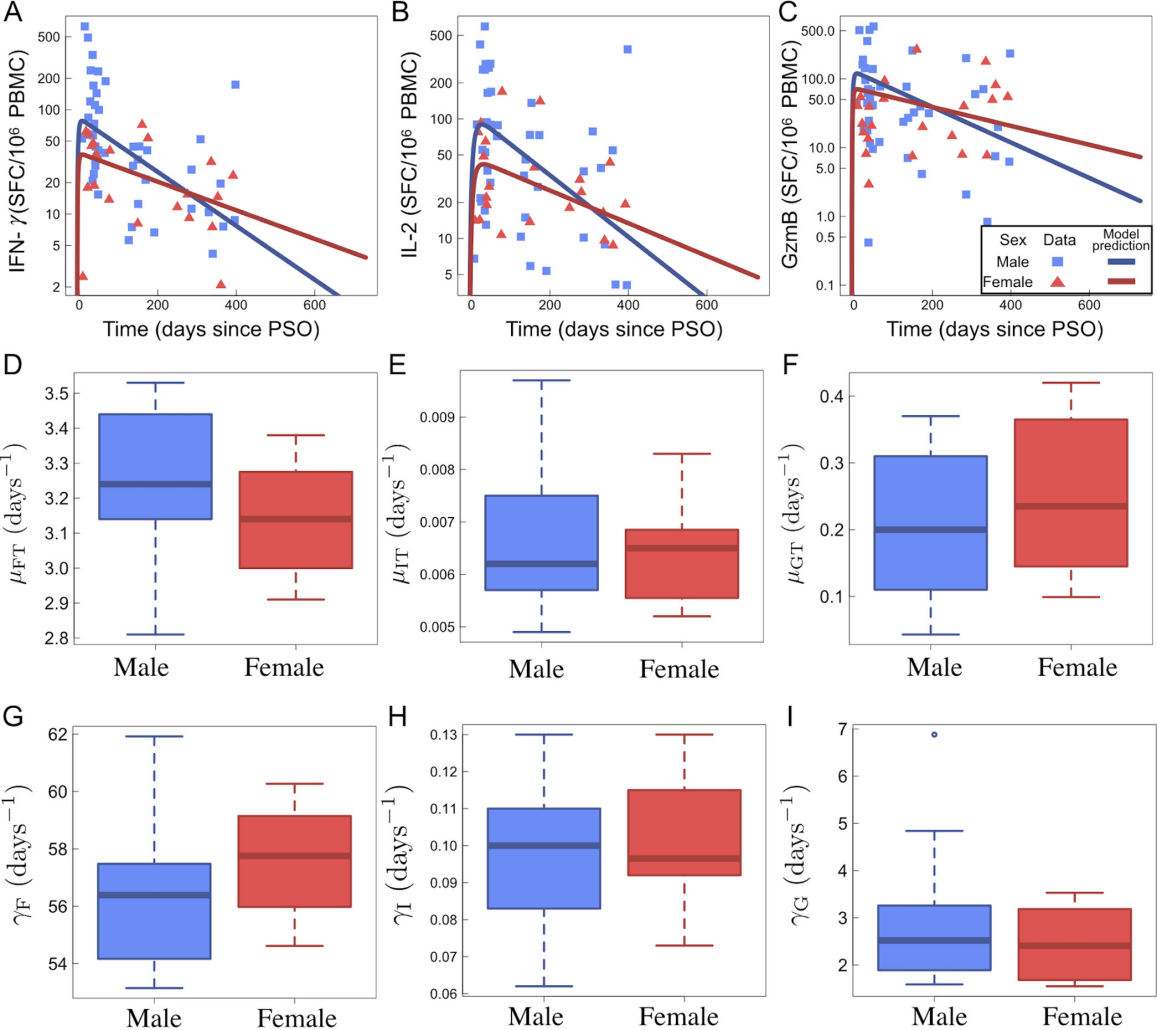
Kinetic model (equations 1 to 8) results sorted by sex. Clinical data in panels A to C are separated by males and females, while model fits are shown as blue and red solid lines. (A) IFN-γ data and fits to equation 7 as a function of days since PSO. (B) IL-2 data and fits to equation 6 as a function of days since PSO. (C) GzmB data and fits to equation 8 as a function of days since PSO. Panels D to I show boxplots of model fitted parameters for equations 6, 7, and 8 classified by sex. (D) IFN-γ stimulation rate by CD4^+^ T cells, μ_FT_, for male and female patients. (E) IL-2 stimulation rate by CD4^+^ T cells, μ_ΙΤ_, for male and female patients. (F) GzmB stimulation rate by CD4^+^ T cells, μ_GT_, for male and female patients. (G) IFN-γ decay rate, γ_F_, for male and female patients. (H) IL-2 decay rate, γ_I_, for male and female patients. (I) GzmB decay rate, γ_G_, for male and female patients.

Using a within-host model (equations 1 to 8) as depicted in Fig. 4, individual fit parameters for the period of data collection for 21 participants were determined and are shown for total SARS-CoV-2 IFN-γ/IL-2/GzmB responses in Fig. 5. The model predicted that the immune response variables peaked at 6 days for IFN-γ, 36 days for IL-2, and 7 days for GzmB. Thus, IFN-γ and GzmB appeared to peak earlier than IL-2. Our model, based on severity of disease, is shown in Fig. 6, detailing the average case severity predicted responses for disease severity as a function of time. For each immune response variable and for each disease severity, we predicted the average response out to 2 years PSO. We found the IFN-γ response to have the highest peak for severe cases, with a value of ~100 SFC/10^6^ PBMC, followed by moderate (~90 SFC/10^6^ PBMC) and then mild (~31 SFC/10^6^ PBMC) disease. For IL-2, all case severities peaked at the same time; however, severe cases displayed a peak response twice that of moderate cases, and moderate disease displayed a peak response 4-fold higher than that of mild cases. In contrast, GzmB displayed little qualitative severity dependence. We then looked at average cytokine production and decay rates with disease severity (Fig. 6D to I). The model predicts that severe illness is associated with reduced IFN-γ and GzmB but increased IL-2 production rates (Fig. 6D to F). For instance, mean IL-2 production (μ_ΙΤ_) rates were found to be 0.0066 ± 0.0010 day^−1^, 0.00685 ± 0.001 day^−1^, and 0.00891 ± 0.00123 day^−1^ for mild, moderate, and severe disease, respectively (Fig. 6E). Mean IL-2 decay (γ_1_) rates were found to be 0.107089 ± 0.015004 day^−1^, 0.0983 ± 0.01608 day^−1^, and 0.0728 ± 0.01303 day^−1^ for mild, moderate, and severe disease, respectively. Mean GzmB production rates were found to be 0.2632 ± 0.117 day^−1^, 0.1623 ± 0.0915 day^−1^, and 0.115 ± 0.0517 day^−1^ for mild, moderate, and severe disease, respectively. We also applied our model based on sex (Fig. 7). We found that for IFN-γ, males displayed faster stimulation and slower decay rates than did females, whereas for GzmB, we found that females displayed faster stimulation and slower decay than did males. For IL-2, stimulation and decay rates were similar between males and females.

**FIG 8:**
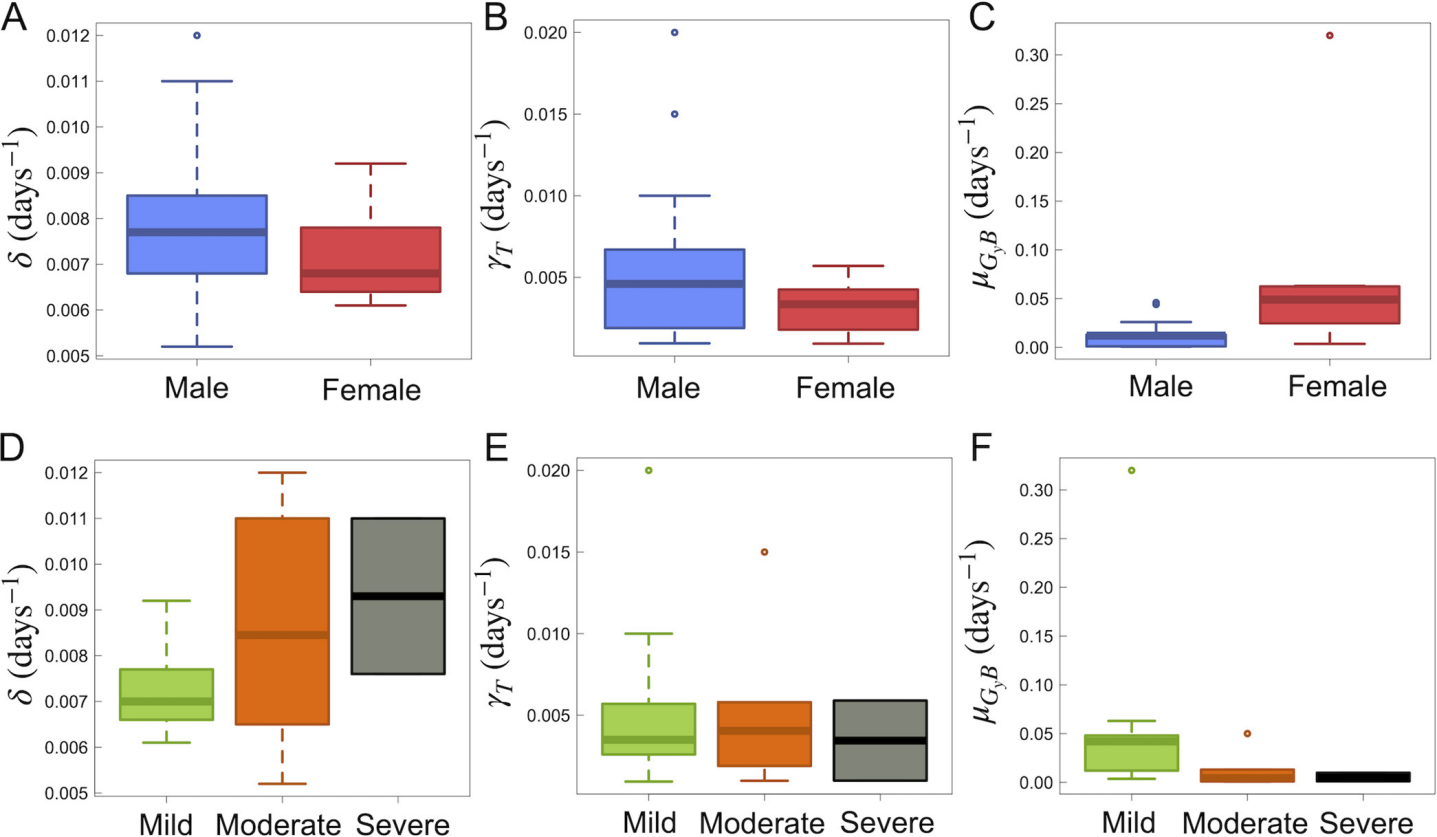
Model (equations 1 to 8) estimated CD4^+^ T cell dynamics and GzmB infected cell killing. Errors are determined by the standard deviation of the respective result across all categorized individuals. (A) CD4^+^ T cell priming rate for males and females. The average rates for males and females were found to be 0.008 ± 0.002 day^−1^ and 0.007 ± 0.001 day^−1^, respectively. (B) Within-host CD4^+^ T cell death rate, γ_T_, for males and females. The average γ_T_ values for males and females were found to be 0.006 ± 0.005 day^−1^ and 0.003 ± 0.002 day^−1^, respectively. (C) Rate of GzmB killing effect of infected target cells, μ_GyB_, for males and females. The average μ_GyB_ values for males and females were found to be 0.015 ± 0.015 day^−1^ and 0.1 ± 0.1 day^−1^, respectively. (D) CD4^+^ T cell priming rate, δ, for mild, moderate, and severe cases. The average δ values for mild, moderate, and severe cases were found to be 0.007 ± 0.001, 0.008 ± 0.002, and 0.009 ± 0.002 day^−1^, respectively. (E) Within-host CD4^+^ T cell death rate, γ_T_, for mild, moderate, and severe cases. The average γ_T_ values for mild, moderate, and severe cases were found to be 0.005 ± 0.005, 0.005 ± 0.005, and 0.003 ± 0.003 day^−1^, respectively. (F) Rate of GzmB killing effect of infected target cells, μ_GyB_, for mild, moderate, and severe cases. The average μ_GyB_ values for mild, moderate, and severe cases were found to be 0.05 ± 0.08, 0.01 ± 0.02, and 0.005 ± 0.006 day^−1^, respectively.

Based on the cytokine and GzmB responses, we were also able to estimate the within-host CD4^+^ T cell half-life from model equation 5. Across all individuals, we found an average CD4^+^ T cell decay (γT) of 0.005 day^−1^. Where we have assumed single exponential decay kinetics for CD4^+^ T cells, this value translates to an average half-life of 139 days (or ~4.6 months). Distributions of CD4^+^ T cell kinetics for males and females as well as individuals with mild, moderate, and severe disease can be found in Fig. 8. Lastly, predictive checks of all cytokines showed all data to fall within 90% confidence intervals for all model fit results (data not shown).

### Proliferative T cell immune responses.

**FIG 9:**
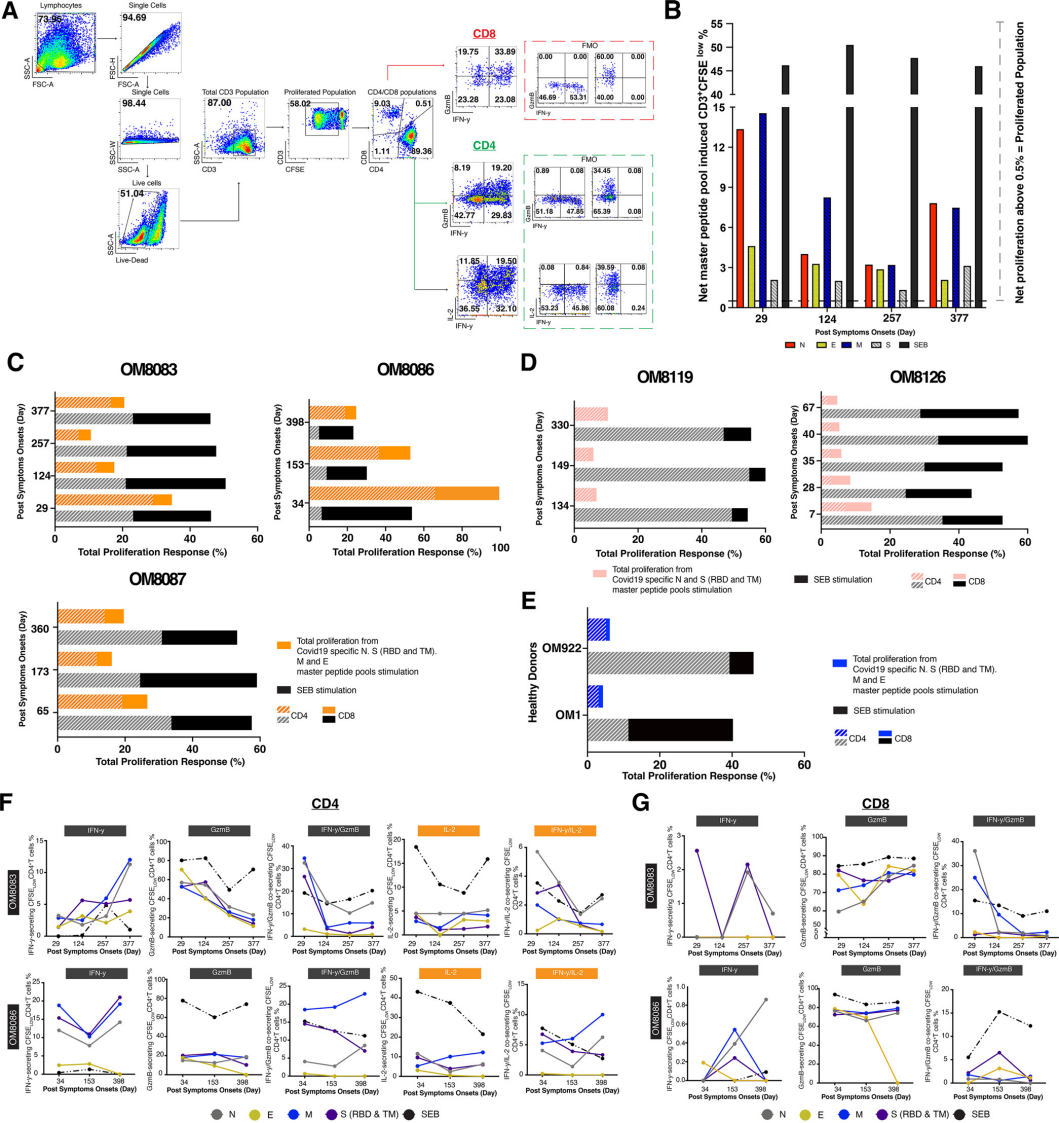
T cell proliferation responses induced by peptide master pools in convalescent COVID-19 subjects over 1 year PSO. (A) Representative flow cytometry gating strategy for CFSE_Low_ CD3^+^ T cells, CD4^+^/CD8^+^ T cells, and IFN-γ/granzyme B/IL-2-producing CD4^+^/CD8^+^ T cells. Fluorescence minus one (FMO) controls for cytokines producing CD4^+^/CD8^+^ T cells were included in the dashed box. (B) Representative net master pool peptides induced T cell proliferative in subject OM8083 at different PSO. (C) Total proliferation from full-set peptide master pool stimulations (N, E, M, S-RBD, and S-TM) in subjects OM8083, OM8086, and OM8087. (D) Total proliferation from full peptide master pools stimulations (N and S-RBD and S-TM) in subjects OM8119 and OM8126. (E) Single-time-point total proliferation response using full set peptide master pools stimulations (N, E, M, S-RBD, and S-TM) in two healthy COVID-19 negative donors, OM1 and OM922. Total proliferation was calculated by adding the total of net peptide master pool-induced CFSE_Low_ responses from all-peptide-master-pool stimulation. The compositions of CD4^+^ and CD8^+^ T cell responses in the total proliferation were calculated by averaging CD4^+^ and CD8^+^ master pool-induced CFSE_Low_ generated at different time points. (F) Percentages of IFN-γ-, GzmB-, IFN-γ/GzmB-, IL-2-, and IFN-γ/IL-2-secreting CFSE_Low_-responding CD4^+^ T cells in subjects OM8083 and OM8086 against days PSO. (G) Percentages of IFN-γ-, GzmB-, and IFN-γ/GzmB-secreting CFSE_Low_-responding CD8^+^ T cell in subjects OM8083 and OM8086 against days PSO.

T cell proliferation in response to viral antigens is important for potent effector and memory responses (24). To further determine virus-specific T cell proliferation capacity over 1 year PSO, five subjects with either severe (OM8083 and OM8086), moderate (OM8087 and OM8126), or mild (OM8119) symptoms were examined using carboxyfluorescein diacetate succinimidyl ester (CFSE)-based flow cytometry analysis over multiple time points (Fig. 9). Notably, all participants had potent T cell proliferative responses to SARS-CoV-2 antigens during the entire period of observation and maintained proliferative capabilities after 1 year PSO regardless of disease severity (Fig. 9B to D). In this study, cross-reactive proliferative responses were also noted in our healthy donors (OM1 and OM922) after stimulation with SARS-CoV-2 peptide master pools, although they tended to be weaker than for the infected individuals (Fig. 9E). The T cell proliferative responses from infected individuals were driven mainly by CD4^+^ T cells, apart from OM8126, after 1 year PSO. This is contrary to the case with our Staphyloccus enterotoxin B (SEB) positive control, where most of the T cell proliferative response were CD8^+^ only or both CD4^+^ and CD8^+^ dominant responses, highlighting the potential downregulation of CD8^+^ T cell responses during SARS-CoV-2 infection. Additionally, the IFN-γ, GzmB, and IL-2 secretion capacities of the CFSE_Low_-responding T cells of subjects OM8083 and OM8086 after 1 year PSO (Fig. 9F and G) were examined via flow cytometry. Both CFSE_Low_-responding CD4^+^ T cells and CFSE_Low_-responding CD8^+^ T cells continually expressed high levels of IFN-γ, IFN-γ/GzmB, IFN-γ/IL-2, or GzmB, further suggesting that convalescent patients developed effective T cells memory response against SARS-CoV-2.

### T cell epitope mapping of SARS-CoV-2 responses.

**FIG 10:**
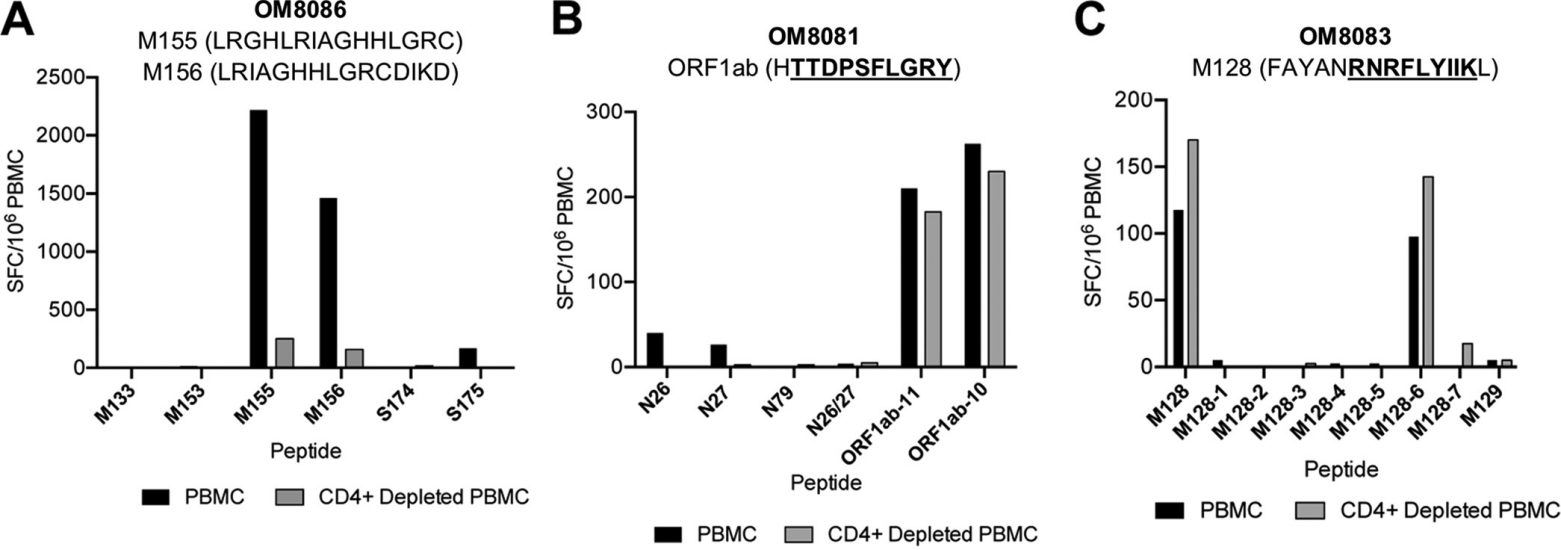
Epitope mapping reveals CD4 dominant responses. Representative graphs of epitope mapping using IFN-γ ELISpot assay with PBMC or CD4-depleted PBMC from three individuals are shown. The strongest peptide per patient is described in the title with the minimal epitope underlined where appropriate. (A) Individual recovered from severe disease; highly responsive to the 15-mers M155 (LRGHLRIAGHHLGRC) and M156 (LRIAGHHLGRCDIKD). (B) Individual recovered from mild disease; highly responsive to the 10-mer ORF1ab-10 (TTDPSFLGRY). (C) Individual recovered from severe disease; highly responsive to the 9-mer M128-6 (RNRFLYIIK). For the 15-mer M128, 9-mers M128-1 to M128-7 were used to find the minimal epitope.

**FIG 11:**
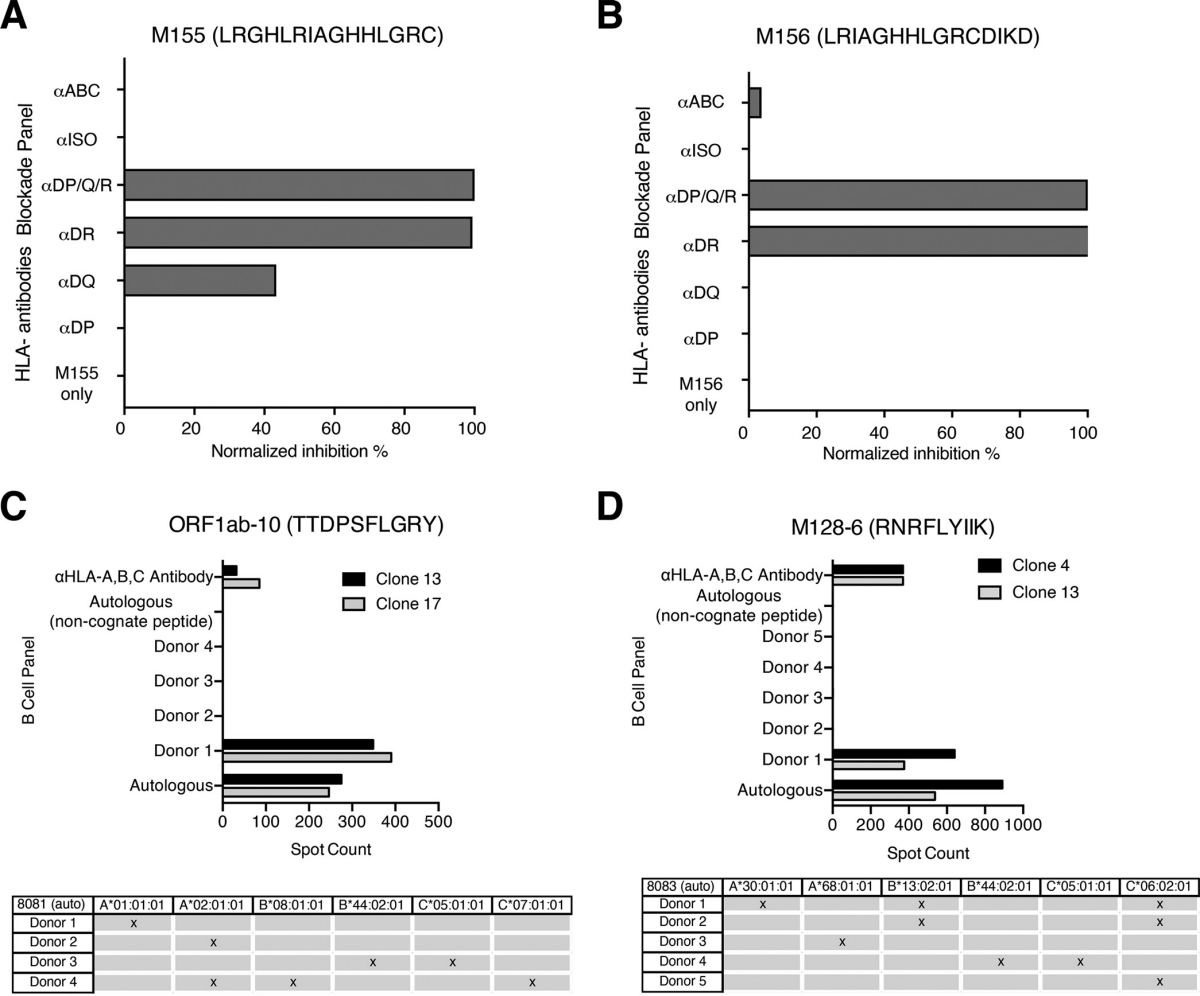
HLA restriction of two CD4^+^ and CD8^+^ epitopes. (A and B) CD4^+^ epitopes M155 and M156 were HLA restricted using anti-HLA-II antibodies on CD4^+^ T cell clones stimulated with autologous B cell lines presenting the cognate peptide. (C and D) CD8^+^ epitopes ORF1ab-10 and M128-6 were HLA restricted using a panel of subject-derived B cell lines. CD8^+^ T cell clones were cocultured with autologous or allogeneic B cells from the panel pulsed with cognate peptide. All responses were measured with IFN-γ ELISpot assay.

In order to further define antigenic regions targeted by CD4^+^ and CD8^+^ T cells, we performed further epitope mapping of IFN-γ responses in 9 individuals (4 with mild, 2 with moderate, and 3 with severe disease) who demonstrated the strongest *ex vivo* IFN-γ cytokine responses. Representative data are shown in Fig. 10, and summary data are shown in Table 2. In some individuals, a substantial frequency of epitope-specific IFN-γ responses could be detected *ex vivo*, varying from 20 SFC/10^6^ PBMC upward to ≥4,000 SFC/10^6^ PBMC. As shown in Table 2, we were able to define a total of 35 epitopes that belong to ORF1ab (1 epitope), N (13 epitopes), M (14 epitopes), and S (7 epitopes). Shorter amino acid epitopes were not included in the total count since they overlap the longer amino acid epitopes. Epitopes from M elicited the strongest response in terms of breadth and frequencies in *ex vivo* PBMC, followed by N, S, and ORF1ab. However, we could not fully characterize the S protein epitopes due to unavailability of full S protein peptide matrices at the time of experimentation. Interestingly, no responses to E were observed. Overall, the majority of epitopes that we could define favored CD4^+^ T cell responses over CD8^+^ T cell responses, with greater breadth in individuals with more severe disease (Table 2). Many of the epitopes we identified were previously identified by others (13, 25–44), suggesting common induction of certain epitopes (Table 2). These included one in the ORF1ab region, the alpha and beta coronavirus families cross-reactive N26-N27 region, the N76-N88 region, the M154-M156 region, and the M160-M163 region (Table 2). Of note is that N26-N27 region was also observed to be cross-reactive in SARS-CoV-1 and the other common cold coronaviruses (HKU1, OC43, NL63, and 229E). The remaining regions (N76-N88, M154-M156, and M160-M163) were characterized in SARS-CoV-1 only. Nine additional epitopes that were not studied as extensively have been further characterized in this study (Table 2). We isolated two CD4^+^ T cell clones from one individual (OM8086). One clone specific for M155 (LRGHLRIAGHHLGRC) was mainly restricted to HLA-DR, although some inhibition (~40%) was observed in the presence of anti-HLA-DQ antibodies (Fig. 11), suggesting some promiscuity of this epitope. The other SARS-COV-2 CD4^+^ T cell clone specific for M156 (LRIAGHHLGRCDIKD) was restricted to HLA-DR only (Fig. 11). Our findings for M155 and M156 were consistent with those of previous studies (12, 13, 28, 29, 33, 45). Specifically, two studies have demonstrated that M155 and M156 were mainly restricted by HLA-DRB1*11, which corresponded to the HLA of our subjects (28, 29) (Table 3). Among the various ORF1ab peptides tested, a response to TTDPSFLGRY was easily detected in 33% of individuals and was determined to be HLA-A*01:01 restricted after testing with a panel of B cell lines (BCL) (Fig. 11C). This immunodominant epitope and HLA restriction were previously reported in other studies (26, 27, 34, 40). We isolated a CD8^+^ T cell clone from one individual recognizing an epitope in the M region, RNRFLYIIK (M128-6), that was restricted to HLA-A*30:01 (Fig. 11). Only *in silico* analyses were conducted for this particular sequence and HLA restriction (25). However, no responses against RNRFLYIIK were observed in two other HLA-A*30:01 patients in our cohort (data not shown). Interestingly, another study had characterized a similar epitope shifted by one amino acid, NRFLYIIKL, to be restricted to HLA-C*07 (34). However, no responses against NRFLYIIKL were detected in three of our HLA-C*07^+^ subjects (data not shown). Although 4/9 of our subjects that were screened for epitope mapping were HLA-A*02:01 restricted, we could not detect any CD8^+^ T cell responses that were restricted to this common allele.

**TABLE 2:** List of identified T cell epitopes^*a*^

Protein	Peptide	Positions	Amino acid sequence	No. of participants positive/tested (%)	*Ex vivo* PBMC, SFC/10^6^ (range)	T cell response	HLA restriction	Reference(s)^*b*^
ORF1ab (*n* = 2)	ORF1ab-10	1637–1646	TTDPSFLGRY	3/9 (33)	65–262.5	**CD8**	HLA-A*01:01	26, 27, 34, 40
	ORF1ab-11	1636–1646	H**TTDPSFLGRY**	3/9 (33)	23–210	**CD8**	HLA-A*01:01	26, 27, 34, 40
Nucleocapsid phosphoprotein (*n* = 14)	N5^*c*^	17–31	FGGPSDSTGSNQNGE	1/9 (11)	33	NA^*d*^		NA
	N15^*c*^	57–71	TQHGKEDLKFPRGQG	1/9 (11)	53	NA		NA
	N26/27 (9-mer)	105–113	SPRWYFYYL	1/9 (11)	47.5	**CD4 + CD8**		12, 13, 26, 28, 30, 35, 40–42
	N26	101–115	MKD**LSPRWYFYYL**GT	2/9 (22)	25–60	**CD4 + CD8**		12, 13, 28, 40, 41
	N27	105–119	**SPRWYFYYL**GTGPEA	2/9 (22)	26.3–75	**CD4 + CD8**		12, 13, 28, 40, 41
	N32	125–139	ANKDGIIWVATEGAL	1/9 (11)	30.5–75	**CD4** + *CD8*		28, 34
	N33	129–143	GIIWVATEGALNTPK	2/9 (22)	25–65	**CD4**		12, 34
	N67	265–279	TKAYNVTQAFGRRGP	1/9 (11)	43	CD4		28
	N76	301–315	WPQIAQFAPSASAFF	2/9 (22)	39.5–47.5	CD4		12, 34, 36, 37
	N79	313–327	AFFGMSRIGMEVTPS	1/9 (11)	96–591.5	CD4/CD8		13, 29, 34, 37, 39
	N81	321–335	GMEVTPSGTWLTYTG	2/9 (22)	25–68	CD4 + CD8		12, 13, 28, 29, 36, 37, 44
	N82	325–339	TPSGTWLTYTGAIKL	1/9 (11)	39.5	CD4 + CD8		13, 29, 32, 34, 37, 44
	N87	345–359	NFKDQVILLNKHIDA	1/9 (11)	89.5	CD4 + CD8		13, 28, 37, 44
	N88	349–363	QVILLNKHIDAYKTF	3/9 (33)	20–637	CD4 + CD8		13, 28, 37, 44
Membrane glycoprotein (*n* = 16)	M128-6 (9-mer)^*c*^	42–50	RNRFLYIIK	1/9 (11)	60–150	*CD8*	HLA-A*30:01	25, 34
	M128^*c*^	37–51	FAYAN**RNRFLYIIK**L	3/9 (33)	42.5–60	*CD8*	HLA-A*30:01	NA
	M129^*c*^	41–55	**NRNRFLYIIK**LIFLW	3/9 (33)	25–95	*CD4/CD8*		25
	M130^*c*^	45–59	FLYIIKLIFLWLLWP	1/9 (11)	36	NA		NA
	M133^*c*^	57–71	LWPVTLACFVLAAVY	1/9 (11)	30	NA		NA
	M135	65–79	FVLAAVYRINWITGG	1/9 (11)	36.5	CD4		28
	M141^*c*^	89–103	GLMWLSYFIASFRLF	2/9 (22)	162.5–545.5	CD4		NA
	M148^*c*^	117–131	NILLNVPLHGTILTR	1/9 (11)	69.5	NA		NA
	M153	137–151	ELVIGAVILRGHLRI	1/9 (11)	44	CD4		13, 28
	M154	141–155	GAVILRGHLRIAGHH	2/9 (22)	36–275	**CD4**		13, 28, 29
	M155/156 (9-mer)	148–156	HLRIAGHHL	1/9 (11)	72	**CD4 **+ CD8		13, 28, 29, 33, 45
	M155	145–159	LRGHLRIAGHHLGRC	3/9 (33)	105–4,630	**CD4 + CD8**	**HLA-DR**	13, 28, 29, 33, 45
	M156	149–163	LRIAGHHLGRCDIKD	3/9 (33)	20–3,214	**CD4 + CD8**	**HLA-DR**	13, 28, 29, 33, 45
	M160	165–179	PKEITVATSRTLSYY	1/9 (11)	95	CD4		13, 28, 33
	M162	173–187	SRTLSYYKLGASQRV	4/9 (44)	20–137.5	**CD4**		13, 28, 29, 33, 34, 38
	M163	177–191	SYYKLGASQRVAGDS	4/9 (44)	39.5–187.5	**CD4 + CD8**		13, 28, 29, 33, 34, 38
Spike Glycoprotein (*n* = 8)	S173	334–348	NLCPFGEVFNATRFA	1/9 (11)	23	CD4		32, 33
	S175	342–356	FNATRFASVYAWNRK	1/9 (11)	36.5–161	**CD4/CD8**		31 – 33
	S176 (9-mer)	349–357	SVYAWNRKR	1/9 (11)	65	**CD4**		32, 33, 39
	S176	346–360	RFA**SVYAWNRKR**ISN	2/9 (22)	153–1,262	**CD4** + *CD8*		32, 33, 39
	S177	350–364	**VYAWNRKR**ISNCVAD	1/9 (11)	182	CD4		32, 33, 39, 44
	S178	354–368	NRKRISNCVADYSVL	1/9 (11)	29–55	**CD4/CD8**		13, 33, 43, 44
	S179	358–372	ISNCVADYSVLYNSA	1/9 (11)	23	CD8		44
	S202	450–464	NYLYRLFRKSNLKPF	1/9 (11)	62.5	**CD4 + CD8**		13, 29, 31–33

a Underlined and bolded epitopes within peptide sequences indicate minimal epitopes confirmed in this study. CD4 or CD8 T cell responses that are bolded and underlined indicate confirmed responses in our study and others, italicized responses indicate confirmed responses in our study and only predicted *in silico* in others, and responses that are not bolded or italicized indicate predicted or confirmed responses in other studies only.

b Reference(s) describing prior described epitopes that elicited IFN-γ responses.

c Novel identified epitope confirmed with T cell IFN-γ ELISpot assays.

d NA, not applicable.

**TABLE 3:** HLA haplotypes of subjects further studied for epitope mapping

Subject	HLA-A	HLA-B	HLA-C	HLA-DPA1	HLA-DPB1	HLA-DQA1	HLA-DQB1	HLA-DRB1	HLA-DRB345
8072	11:01	40:01	03:04	02:01	02:01	01:03	04:01	04:05	DRB4*01:03
			07:02	02:02	13:01	03:03	06:01	08:03	
8073	29:01	13:02	04:01	01:03	04:01	02:01	02:02	07:01	DRB3*02:02
	30:01	35:03	06:02	01:03	04:02	05:05	03:01	11:04	DRB4*01:03
8076	11:01	52:01	06:02	01:03	04:02	01:01	05:01	01:01	DRB5*02:02
	68:02	53:01	12:02	02:02	13:01	01:02	05:02	16:02	
8078	01:01	40:01	03:04	01:03	02:01	03:01	03:01	04:01	DRB4*01:03
	02:01	44:02	05:01		04:01	03:03	03:02	04:04	DRB4*01:03
8081	01:01	08:01	05:01	01:03	04:01	01:02	02:01	03:01	DRB3*01:01
	02:01	44:02	07:01	02:06	05:01	05:01	06:02	15:01	DRB5*01:01
8083	30:01	13:02	05:01	01:03	02:01	01:03	02:02	07:01	DRB3*02:02
	68:01	44:02	06:02		04:01	02:01	06:03	13:01	DRB4*01:03
8086	24:02	07:05	03:03	01:03	02:01	01:05	03:01	10:01	DRB3*02:02
	32:01	15:01	15:05		04:02	05:05	05:01	11:03	
8087	02:01	07:02	07:02	01:03	02:01	05:01	02:01	03:01	DRB3*01:01
	32:01	41:01	17:01		03:01				DRB3*02:02
8099	02:01	35:08	04:01	01:03	13:01	03:01	03:02	04:03	DRB4*01:03
	30:01	49:01	07:01	02:01	14:01	03:03	04:02	04:04	DRB4*01:03

### CD4^+^ and CD8^+^ SARS-CoV-2-specific T cells can recognize and kill target cells.

**FIG 12:**
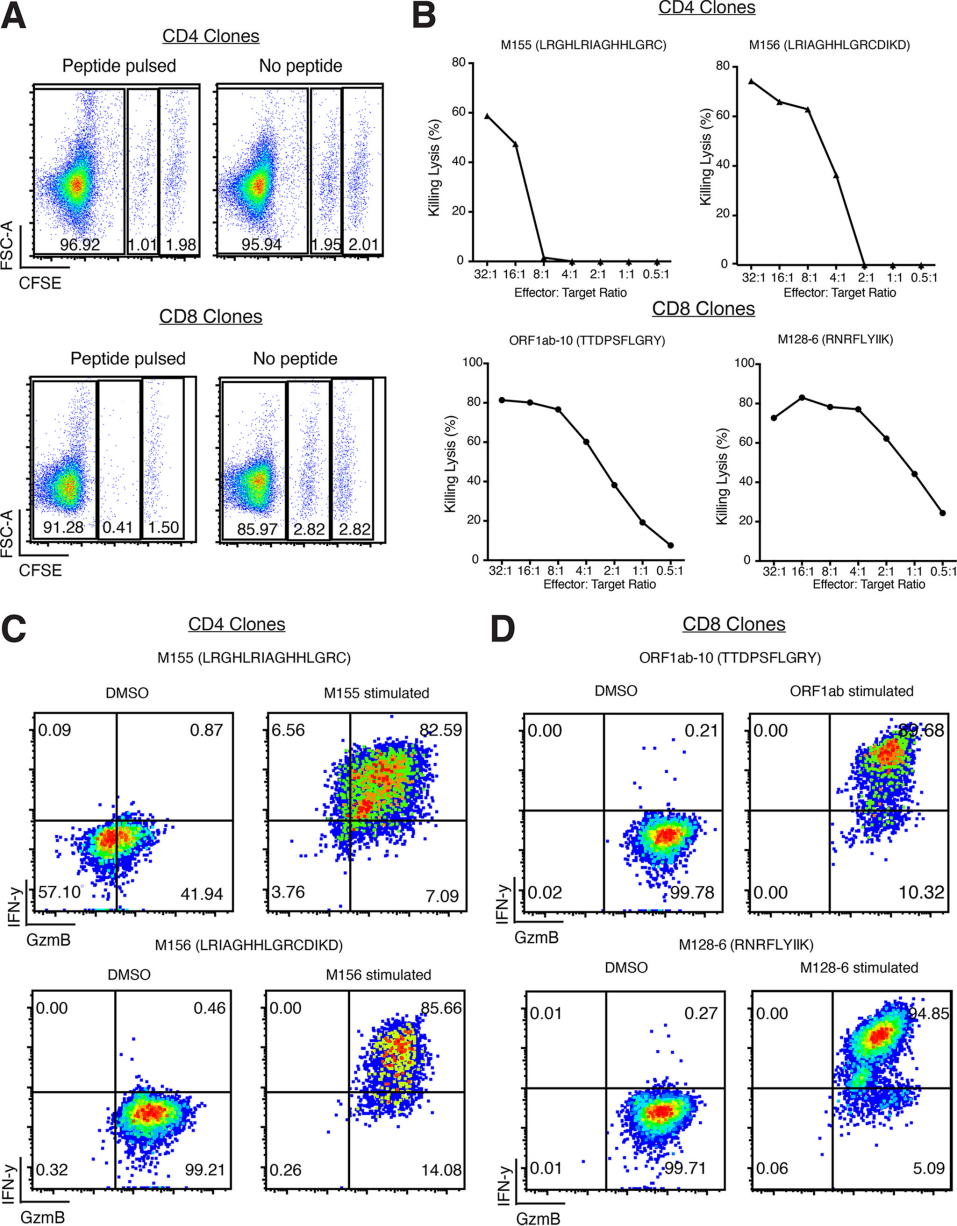
Assessment of recognition and killing capabilities of SARS-CoV-2 membrane/ORF1ab-specific T cell clones. (A) Representative recognition and killing capabilities of SARS-COV-2-specific CD4^+^ and CD8^+^ T cell clones against peptide-pulsed, CFSE_Low_ target cells. (B) Percentage killing lysis at different effector/target ratios of CD4^+^ and CD8^+^ T cell clones. (C and D) Flow cytometry analysis of intracellular cytokines secretion of IFN-γ and GzmB by CD4^+^ and CD8^+^ T cell clones in the presence of peptide-pulsed target cells.

Both SARS-CoV-2-specific CD4^+^ and CD8^+^ T cell clones were able to kill peptide-pulsed autologous B cell line target cells (Fig. 12A and B). Interestingly, SARS-CoV-2 CD8^+^ T cell clones tended to have enhanced cytotoxic capabilities compared to the SARS-CoV-2 CD4^+^ T cell clones since they could kill at lower effector/target cell ratios (Fig. 12B). Both CD4^+^ and CD8^+^ T cell clones elicited strong IFN-γ and GzmB responses when stimulated with their specific peptide of interest (Fig. 12C and D).

## DISCUSSION

The establishment of cell-mediated immunity is critical for long-lasting immunity against most viral infections, including coronaviruses. After SARS-CoV-1 infection, antibody-mediated immunity was seen to wane over time (46, 47); however, SARS-CoV-1-specific memory T cells were observed to persist for 6 to 11 years postinfection in several studies, and these cells were at higher frequencies in those with more severe illness (36, 37, 46, 47). In this study, we characterized cytokine-producing T cell responses to SARS-CoV-2 structural proteins during acute infection and found potent Th1-focused pluri-cytokine-producing responses early and during convalescence in SARS-CoV-2 survivors, with frequencies ranging from 100 to >1,000 cytokine-producing cells per 10^6^ PBMC. These frequencies are similar to those reported by Le Bert et al. (12), who reported IFN-γ ELISpot assay against N for SARS-CoV-2-infected individuals. Our data expands these findings to include IL-2, GzmB, and IFN-γ toward all structural proteins. It should be noted that previous estimates describing SARS-CoV-2-specific T cells measured higher frequencies of antigen-specific cells when using *ex vivo* flow cytometric techniques based on activation marker upregulation when exposed to peptide pools (14, 22, 48). However, these frequencies may not necessarily reflect the circulating numbers of antigen-specific cells that are capable of producing cytokines. A number of conclusions can be made from our observations which may, in part, be unique to SARS-CoV-2. The SARS-CoV-2 T cell response preferentially induces GzmB and IL-2 over IFN-γ; CD4^+^ T cell responses make up the majority of this response; T cell responses favored N, S, and M but not the E structural proteins; and more severe disease elicited higher memory responses in survivors. The role of GzmB in inducing a potent response is likely important for viral clearance, as previous studies have shown a role for granzymes in control of viral infections in humans, such as acute HIV (20, 49). Although virus infections generally induce very potent CD8^+^ T cell expansions, this was not observed in our and others’ cohorts for SARS-CoV-2 infection, though it was observed for SARS-CoV-1 (37, 47, 48, 50). The reasons for this are currently unclear, although a recent study suggests that SARS-CoV-2 ORF8 protein may interfere with major histocompatibility complex (MHC) class I presentation, thus possibly preventing conventional CD8^+^ T cell priming (51). A potential caveat of our data is that although we used a nonbiased approach to detect responding T cells with peptide pools, the peptides were 15-mers in length and could have potentially not been sufficient to induce cytokine responses to embedded 9-mer epitopes within the 15-mers in some CD8^+^ T cell populations.

Our data showed a number of features of the kinetics of SARS-CoV-2 T cell immunity. The mathematical modeling results showed early induction of the effector cytokines IFN-γ and GzmB, with IL-2 induced later, which is consistent with the effector role of IFN-γ/GzmB in viral clearance and IL-2 to induce long-term memory. Modeling also showed that disease severity induces greater IFN-γ/IL-2, consistent with increased antigen load during moderate and severe disease. The similar production and decay rates of GzmB in mild disease suggest that GzmB may have greater importance at viral clearance, resulting in a milder disease course. Our decay rates of T cell cytokine responses were similar to those of Dan et al. (14), who observed a half-life decay of 3 to 5 months, while we predicted 4.6 months. It should be noted that we only modeled antigen-specific responses in peripheral blood, and our modeling studies cannot assess whether cells have migrated out of blood into tissues and undergo antigen-specific expansion there. Our modeling studies also indicated that males and females may differ in IFN-γ and GzmB production in median and variance during infection. Whether these findings help explain the increased mortality rates in men over women from COVID-19 illness will require further study (52, 53). We followed our cohort for at least 1 year and, encouragingly, still found persistence of T cell immunity in the majority of individuals, in particular, SARS-CoV-2-specific IL-2 in 86% and IFN-γ/GzmB in about 70%. Importantly, we were able to find potent proliferative T cell responses that produced cytokines even at the last time points in all individuals studied. These findings indicate that although decay of T cell immunity can be observed over 1 year, one can still induce potent proliferative and effector cytokine responses in T cells obtained 1 year after infection in most, if not, all individuals after restimulation. The role that these responses play in protection against infection and/or disease will require further study and follow-up.

SARS-CoV-2 T cell epitope discovery will inform future T cell-based coronavirus vaccines, particularly in the setting of antibody escape variants. Several putative epitopes found in SARS-CoV-1 were, interestingly, observed in SARS-CoV-2. Specifically, the identification of similar epitopes in the N26-N27, N76-N88, and M154-M163 regions in both SARS-CoV-1 and SARS-CoV-2 and in other coronavirus families suggest that the common induction of certain epitopes across coronavirus families could be used for vaccine development. We have also described a number of new epitopes that could be incorporated in T cell vaccine designs. For example, the M128-6 epitope (RNRFLYIIK) was a CD8^+^ HLA*30:01-restricted epitope. In addition, we observed cross-reactive T cell responses in a large number of uninfected individuals which likely reflect previous infection with seasonal coronaviruses. Recent work suggests that these cross-reactive responses may be important in alleviating, rather than exacerbating, disease caused by SARS-CoV-2 (54). The identification of additional, novel immunodominant epitopes, such as M128-6 and M141, and the demonstration of recognition and cytotoxic capabilities of CD8^+^ and CD4^+^ T cell clones in the M region will be critical for next-generation vaccines against potential spike antibody escape variants. Of the epitopes induced on our subjects, a few mutations were observed in the omicron variant (A63T for peptide M133, deletion of residue 31 for peptide N5 in BA.1, and S371F for peptide S170 in BA.2 [Table 2]). Further work will determine if these mutations confer escape from the T cell responses. Despite the evasion of CD8^+^ T cell response induction, we found that CD4^+^ T cell clones can kill peptide-pulsed target cells, albeit at seemingly lower effector/target cell ratios than the CD8^+^ T cell clones that we isolated. These features may reflect one reason why SARS-CoV-2 can induce severe disease in a subset of individuals. A combination of a poor CD8^+^ T cell response, with greater killing capacity, and an overexuberant CD4^+^ T cell responses may contribute to disease severity. Thus, further studies will be needed to determine the relative functional avidity of CD4^+^ versus CD8^+^ T cells targeting SARS-CoV-2.

In conclusion, we show that SARS-CoV-2-specific memory T cells remain detectable after 1 year PSO and are capable of proliferating and generating IFN-γ, IL-2, and GzmB responses against SARS-CoV-2 structural proteins, which will likely influence disease course during reinfection with variants and may help explain superior responses to vaccines.

## MATERIALS AND METHODS

### Human subjects and study approval.

Healthy subjects and individuals with COVID-19 infection as diagnosed by positive nasopharyngeal SARS-CoV-2 PCR were recruited, and informed consent was obtained from them for blood draws and/or leukapheresis through an research ethics board (REB)-approved protocol (St. Michael’s Hospital/Unity Health, Toronto, Canada; REB20-044). Asymptomatic COVID-19-negative controls had no history of viral infection and negative serology (IgG) for SARS-CoV-2 full spike (S), spike receptor binding domain (RBD), and nucleocapsid (N) proteins by enzyme-linked immunosorbent assay (ELISA) as previously described by members of our group (55). All human subject research was done in compliance with the Declaration of Helsinki.

### PBMC isolation.

Whole blood or leukapheresis samples were acquired from St. Michael’s Hospital, Unity Health. Peripheral blood mononuclear cells (PBMC) were isolated by centrifugation using Ficoll-Paque PLUS (GE Healthcare). PBMC were resuspended in R10 medium consisting of RPMI 1640 (Wisent), 10% heat-inactivated fetal bovine serum (FBS; Wisent), 10 mM HEPES (Wisent), 2 mM l-glutamine (Wisent), and 100 U of penicillin-streptomycin solution (Wisent). PBMC were diluted 1:1 with freezing medium consisting of 20% dimethyl sulfoxide (DMSO; Sigma-Aldrich) in FBS and aliquoted for −150°C storage. PBMC used in the T cell epitope mapping were sent to Scisco Genetics Inc. (Seattle, WA) for HLA typing (Table 3).

### SARS-CoV-2 peptides and peptide pool synthesis.

SARS-CoV-2 15-mer peptides overlapping by 11 amino acids from the four main structural proteins of the SARS-CoV-2 reference sequence (GenBank accession number NC_045512.2) were synthesized by GenScript (Piscataway, NJ) for T cell epitope mapping. In total, 212 individual peptides containing 102 nucleocapsid, 12 envelope (E), 49 membrane (M), and 49 spike 15-mers were synthesized. The S 15-mers used for the T cell epitope mapping spanned only the receptor binding domain (RBD) and transmembrane domain (TM) due to cost considerations. Using these peptides, 30 matrix peptide pools containing 19 to 23 15-mers were generated using the Deconvolute This! program version 1.0 (56, 57). For longitudinal assessment of participants’ cytokine responses, 4 peptide master pools for each structural protein (N, E, M, and S-RBD plus S-TM) and 2 peptide master pools containing the full S protein (S1 and S2) were synthesized by GenScript and JPT (Berlin, Germany), respectively.

### *Ex vivo* ELISpot assay.

*Ex vivo* ELISpot assays were performed using human IFN-γ, IL-2, and granzyme B (GzmB) antibodies (Mabtech). Briefly, MSIPS4W plates (Millipore) were activated with 35% ethanol and coated with primary antibody: 10 μg/mL for IFN-γ (1-D1K), 10 μg/mL for IL-2 (MT2A91/2C95), and 15 μg/mL for GzmB (MT28). Plates were incubated overnight at 4°C, washed with phosphate-buffered saline (PBS), and blocked with R10 for 1 h at 37°C. PBMC were thawed and rested for at least 2 h prior to plating. Plates were washed and PBMC were plated at 2 × 10^5^ cells/well. Peptides were added at a final concentration of 1 μg/mL. Plates were incubated at 37°C for 24 h (IFN-γ and IL-2) or 48 h (GzmB). After incubation, plates were washed with PBS containing 0.05% Tween 20 (BioShop) before the addition of human IFN-γ (7-B6-1), IL-2 (MT8G10), and/or GzmB (MT8610) biotinylated secondary antibodies. For dual-color assays, alkaline phosphatase (ALP) conjugated to IFN-γ secondary antibodies were used with other biotinylated secondary antibodies targeting another cytokine. Streptavidin-ALP and/or streptavidin-horseradish peroxidase (HRP) was added after washing, and spots were developed using Vector Blue for ALP and/or Vector NovaRED for HRP (Vector Laboratories). All conditions were done in duplicate unless otherwise stated. Spots were quantified with an ImmunoSpot S3 analyzer (Cellular Technology Limited).

For data analysis, mean spots of the negative-control wells (DMSO) were subtracted from all peptide-stimulated wells before results were normalized to spot-forming cells per million (SFC/10^6^) PBMC. For T cell cloning and mapping studies, results were considered positive if wells were ≥20 SFC/10^6^ PBMC and twice the mean value of negative-control wells.

### Generation of immortalized BCL.

B cell lines (BCL) were generated using PBMC and immortalized with Epstein-Barr virus (EBV). Frozen PBMC were thawed and incubated with 2.5 mL of supernatant from B95-8 cell lines for 2 h in a 37°C water bath. A total of 1 μg/mL of cyclosporine in 5 mL of R10 was then added to the cell suspension and incubated in a T25 flask for 3 weeks at 37°C. B cells that were difficult to immortalize in this manner were first purified using a human B cell isolation kit (STEMCELL) and then incubated with B95-8 supernatant.

### Generation of SARS-CoV-2-specific CD4^+^/CD8^+^ T cell clones and lines.

Frozen PBMC were thawed and first depleted using CD4^+^ or CD8^+^ positive selection kits (STEMCELL). Depleted PBMC were incubated at 37°C overnight with the peptide of interest at 10 μg/mL in R5 medium that contained 5% human serum instead (Wisent). After overnight incubation, SARS-CoV-2 peptide-specific CD4^+^ T cells were selected using an IL-2 secretion kit (Miltenyi), while SARS-CoV-2 peptide-specific CD8^+^ T cells were obtained using an IFN-γ secretion kit according to the manufacturer’s instructions (Miltenyi). Enriched T cells were resuspended in R10-Max medium that contained 2 mM GlutaMAX instead (Gibco). T cells were plated in 96-well plates at limiting dilution. T cells were then cocultured with irradiated feeder cells containing allogeneic PBMC and BCL, 50 U/mL of IL-2 (R&D Systems), 10 ng/mL of IL-15 (R&D Systems), and 25 ng/mL of mouse anti-human CD3 antibody (UCHT1; BD Bioscience). T cells were incubated at 37°C for 4 to 5 weeks and fed biweekly with R10-Max and 50 U/mL of IL-2. Next, IFN-γ ELISpot assay was used to screen the potential T cell clones for specificity against the peptide of interest (1 μg/mL) in the presence of autologous BCL (1 × 10^3^ cells/well). For T cell lines, PBMC were pulsed with 50 μg/mL of the target peptide for 1 h at 37°C, diluted to 1 μg/mL of peptide, and plated in 12-well plates at 2 × 10^6^ cells/mL. After 24 h of incubation at 37°C, 50 U/mL of IL-2 and 25 ng/mL of IL-7 were added. PBMC were kept in culture for 3 to 5 weeks and stimulated weekly with 1 μg/mL of target peptide, 50 U/mL of IL-2, and 25 ng/mL of IL-7. IFN-γ ELISpot assay was then used to test the specificity of potential T cell lines against the target peptide (1 μg/mL) in the presence of autologous BCL (1 × 10^3^ cells/well). The T cell compositions of clones and lines that elicited positive IFN-γ ELISpot responses were then examined via flow cytometry.

### HLA restriction of T cell clones and lines.

To determine HLA I-restricted epitopes from CD8^+^ T cells, a B cell panel containing autologous and allogeneic BCL was used. BCL were pulsed with the specific peptide for 1 h at 10 μg/mL, washed, and then cocultured with CD8^+^ T cell clones in an IFN-γ ELISpot assay. To determine HLA II-restricted epitopes from CD4^+^ T cell clones or lines, autologous BCL were blocked with 10 μg/mL of either anti-HLA-A/B/C (BD Biosciences), anti-HLA-DP (Abcam), anti-HLA-DQ (BioLegend), anti-HLA-DR (BioLegend), or anti-HLA-DP/DQ/DR (BioLegend) antibodies for 30 min and then pulsed with the specific peptide for 1 h at 10 μg/mL. Pulsed BCL (1 × 10^3^ cells/well) were then washed 3 times with R10, mixed with CD4^+^ T cells (1 × 10^4^ to 2 × 10^4^ cells/well), and tested in IFN-γ ELISpot assay in the presence of 10 μg/mL of anti-HLA antibodies.

### CFSE T cell proliferation assay.

Carboxyfluorescein diacetate succinimidyl ester (CFSE) T cell proliferation assay was performed as previously described (58). Briefly, PBMC obtained from various time points were prelabeled with 5 μM CFSE (Thermo Fisher Scientific) in PBS with 2.5% FBS for 8 min in a 37°C water bath. CFSE-labeled cells were then resuspended in R10 medium supplemented with recombinant IL-2 (R&D Systems) and 2-mercaptoethanol (Thermo Fisher Scientific) before being plated in 96-well U-bottom plates at 4 × 10^5^ cells/well. PBMC were then prestimulated with 0.1 μg of N, E, M, and S (RBD and TM) master peptide pools, DMSO (negative control), or SEB (positive control) and incubated at 37°C for 5 days. On day 6, PBMC were restimulated with 1 μg/mL of master peptide pools in the presence of brefeldin A (GolgiPlug; BD Bioscience) and monensin (GolgiStop; BD Bioscience) for 24 h. On day 7, PBMC were first stained with LIVE/DEAD fixable blue dead cell stain (Thermo Fisher Scientific) and incubated with Fc receptor blocking solution (human TruStain FcX; BioLegend) before surface staining with fluorochrome-conjugated antibodies to CD3 (allophycocyanin [APC]-Cy7; clone SK7), CD4 (peridinin chlorophyll protein [PerCP]-Cy5.5; clone SK3), and CD8 (phycoerythrin [PE]; clone HIT8a). Cells were then fixed using BD Cytofix/Cytoperm and permeabilized using BD Perm/Wash according to the manufacturer’s protocol before staining with anti-IFN-γ (APC; clone 4S.B3), anti-GzmB (BV421; clone GB11), and anti-IL-2 (BV711; clone MQ1-17H1). All antibodies were purchased from BD Bioscience. Samples were then acquired on a BD Fortessa-X20 instrument. Net master peptide pool-induced CFSE_Low_ responses were calculated as the percentage of master peptide pools with reduced CFSE fluorescence minus the percentage of control (DMSO) stimulated cells with reduced CFSE fluorescence.

### CTL killing assay.

Cytotoxic T lymphocyte (CTL) killing assays were performed as previously described (59), with minor modifications. Autologous BCL were used as target cells and preincubated with 40 ng/mL of IFN-γ (R&D System) for 18 h at 37°C. To differentiate peptide-pulsed and non-peptide-pulsed target cell populations, BCL were stained with 0.02 μM CFSE (CFSE_Low_) or 0.2 μM CFSE (CFSE_High_) for 15 min at 37°C, respectively. CFSE_Low_ BCL were then pulsed with the target peptide at 5 μg/mL for 45 min at 37°C. Both CFSE_Low_ and CFSE_High_-labeled BCL were washed twice with warm R10 prior to mixing at a 1:1 ratio and resuspended to 2 × 10^5^ cells/mL for plating. For effector cells, T cell clones were washed three times with warm R10 to remove any excess cytokines and serially diluted from 32:1 to 0.5:1 (effector/target ratio). Both target and effector cells were then plated to a 96-well U-bottom plate and incubated for 6 h at 37°C. Cells were then stained with LIVE/DEAD fixable near IR cell stain (Thermo Fisher Scientific) and incubated with Fc receptor blocking solution (human TruStain FcX; BioLegend) before surface staining with fluorochrome-conjugated antibodies to CD4 (BV711; clone SK3) and CD8 (PE; clone HIT8a). In a separate experiment, intracellular cytokines secreted by the T cell clones were determined as previously described by using anti-IFN-γ (APC; clone 4S.B3) and anti-GzmB (BV421; clone GB11). All antibodies were purchased from BD Bioscience. Samples were then acquired on the BD LSR Fortessa.

### Experimental software and statistical analysis.

All flow cytometry data were analyzed using FlowJo 10.8.0 software (Treestar, Ashland, OR). Prism 9.0 (GraphPad, San Diego, CA) was used to perform statistical and graphical analyses.

### Within-host model for SARS-CoV-2 infection and subsequent immune response.

To model the initial infection and subsequent production of infectious virions, we used a target cell-limited model with an eclipse phase (equations 1 to 4; full model depicted and described in Fig. 4) similar to that successfully used to model influenza A virus (60, 61), as well as SARS-CoV-2 (62). Our full model is given by equations 1 to 8. The equation for healthy target cells is

(1) $$dy_{H}/\mathrm{dt}\;=\;-\beta y_{H}v$$

The equation for the eclipse stage is

(2) $$dy_{E}/\mathrm{dt}\;=\beta y_{H}v\;-\;\left(D\;+\;E\right)y_{E}$$

The equation for budding cells is

(3) $$dy_{B}/\mathrm{dt}\;=\;Ey_{E}-\;Dy_{B}-\mu_{\mathrm{GyB}}y_{B}G$$

The equation for infectious virions is

(4) $$\mathrm{dv}/\mathrm{dt}\;=\;\mathrm{eB}y_{B}-\;\beta y_{H}v\;-\;\mathrm{Cv}$$

The equation for primed CD4^+^ T cells is

(5) $$\text{dT}/\text{dt }=\delta y_{\text{B}}+\alpha_{\text{TI}}(\text{I}/\left[s_{\text{I}}+I]\right)T+\alpha_{\text{TF}}\left(F/\left[s_{F}+\;F\right]\right)-\gamma_{\text{T}}\text{T}$$

The equation for IL-2 is

(6) $$\mathrm{dI}/\mathrm{dt}=\mu_{\mathrm{IT}}T\;-\alpha_{\mathrm{IT}}IT\;-\gamma_{I}I\;\;\;\;\;\;\;\;\;\;$$

The equation for IFN-γ is

(7) $$\text{dF}/\text{dt }=\mu_{\text{FT}}\text{T }-\alpha_{\text{FT}}\text{FT }-\gamma_{\text{F}}\text{F}$$

The equation for GzmB is

(8) $$\mathrm{dG}/\mathrm{dt}\;=\mu_{\mathrm{GT}}T\;-\gamma_{G}G$$

### Within-host model parameter estimation and fitting assessment.

All fits to clinical data using our model (equations 1 to 8) were performed in Monolix (version 2020R1) using nonlinear mixed-effects models. Individual parameters (Tables 4 and 5) for each data set were determined by the maximum likelihood estimator Stochastic Approximation Expectation–Maximization (SAEM), and all fits met the standard convergence criteria (complete likelihood estimator). As shown in Table 4, many parameters from equations 1 to 8 are fit for this study. However, parameters from equations 1 to 4 were chosen based on previous studies on SARS-CoV-2.

**TABLE 4:** Model parameter definitions, population (*n* = 21) fits, and average male and average female fit values

Parameter	Definition	Full cohort (*n* = 21)	Comment	Avg male response (*n* = 13)	Avg female response (*n* = 8)
β	Per target cell attachment rate	1.5 × 10^−5^	Reference 62	1.39 × 10^−5^	1.49 × 10^−5^
D	Target cell death rate	0.33	Reference 62	0.37	0.4
E	Eclipse rate	4	Reference 62	4.1	3.71
B	Infected cell budding rate	1,115,038	Reference 62	1.06 × 10^7^	1.11 × 10^7^
C	Virion clearance rate	0.76	Fixed	0.78	0.59
δ	Rate of CD4^+^ T cell priming	0.0083	Fit	0.0082	0.0072
$\alpha_{\text{TI}}$	CD4^+^ T cell priming enhancement due to IL-2	0.000003	Fit	2.96 × 10^−6^	2.85 × 10^−6^
s_I_	CD4^+^ duplication threshold due to IL-2	593	Fixed	593	593
$\alpha_{\text{TF}}$	CD4^+^ T cell priming enhancement due to IFN-γ	1.2 × 10^−6^	Fit	1.22 × 10^−6^	1.23 × 10^−6^
s_F_	CD4^+^ T cell duplication threshold due to IFN-γ	627	Fixed	627	627
$\gamma_{\text{T}}$	CD4^+^ T cell death rate	0.001 to 0.7	Fixed range	0.0061	0.0032
$\mu_{\text{IT}}$	IL-2 stimulation rate by CD4^+^ T cells	0.0065	Fit	0.0066	0.0064
$\alpha_{\text{IT}}$	IL-2 clearance by CD4^+^ T cells	4.9 × 10^−7^	Fit	4.83 × 10^−7^	4.96 × 10^−7^
$\gamma_{\text{I}}$	IL-2 natural degradation rate	0.096	Fit	0.1	0.1
$\mu_{\text{FT}}$	IFN-γ stimulation rate by CD4^+^ cells	3.21	Fit	3.23	3.14
$\alpha_{\text{FT}}$	IFN-γ clearance by CD4^+^ T cells	0.00022	Fit	0.0002	0.0002
$\gamma_{\text{F}}$	IFN-γ natural degradation rate	56.57	Fit	56.57	57.58
$\mu_{\text{GT}}$	GzmB stimulation rate by CD4^+^ cells	0.18	Fit	0.2	0.25
$\gamma_{\text{G}}$	GzmB natural degradation rate	2.45	Fit	2.93	2.46
ε	Fraction of infection virions	0.001	Reference 62	0.001	0.001
$\mu_{\text{GyB}}$	Rate of GzmB-infected cell killing	0.0079	Fit	0.014	0.074
BIC	Bayesian information criteria	2,258	Fit		
AIC	Akaike information criteria	2,219	Fit		

**TABLE 5:** Individual kinetic model (equations 1 to 8) fit parameters

Subject	δ	α_TI_	α_TF_	γ_T_	u_IT_	α_IT_	γ_I_	u_FT_	α_FT_	γ_F_	u_GT_	γ_G_	u_GyB_
OM8072	0.011	0.000003	0.0000013	0.015	0.0068	0.00000047	0.093	3.23	0.00022	56.39	0.31	1.71	0.00076
OM8074	0.0068	0.0000034	0.0000012	0.0046	0.0049	0.00000045	0.13	3.51	0.00021	53.53	0.2	2.52	0.046
OM8076	0.0073	0.0000029	0.0000013	0.01	0.0061	0.00000048	0.11	3.15	0.00022	57.37	0.31	1.9	0.026
OM8077	0.0062	0.0000028	0.0000013	0.0029	0.0061	0.00000056	0.1	3.44	0.00021	54.17	0.043	6.88	0.012
OM8078	0.0066	0.0000031	0.0000012	0.0057	0.0067	0.00000046	0.096	3.15	0.00021	57.64	0.12	3.44	0.063
OM8081	0.0078	0.0000029	0.0000012	0.0067	0.0062	0.00000045	0.1	3.32	0.00022	55.27	0.11	3.26	0.012
OM8083	0.0076	0.0000028	0.0000012	0.0059	0.0097	0.00000045	0.062	2.81	0.00022	61.92	0.073	4.02	0.0098
OM8084	0.007	0.0000026	0.0000014	0.0028	0.0055	0.00000055	0.12	3.25	0.00022	56.43	0.42	1.65	0.048
OM8086	0.011	0.000003	0.0000012	0.001	0.008	0.0000004	0.081	3.14	0.00022	57.48	0.14	2.76	0.0011
OM8087	0.0052	0.0000028	0.0000012	0.0019	0.0075	0.00000045	0.083	2.99	0.00022	59.87	0.057	4.84	0.013
OM8088	0.0085	0.0000029	0.0000012	0.02	0.0057	0.00000052	0.11	3.34	0.00021	55	0.27	1.95	0.0054
OM8094	0.0085	0.0000028	0.0000013	0.0039	0.0083	0.00000047	0.073	2.91	0.00022	60.27	0.17	2.54	0.0076
OM8096	0.012	0.000003	0.0000012	0.0058	0.0063	0.0000006	0.097	3.53	0.0002	53.15	0.12	2.95	0.00096
OM8097	0.0071	0.000003	0.0000012	0.00094	0.0052	0.00000049	0.13	3.3	0.00022	55.53	0.42	1.55	0.042
OM8100	0.0063	0.0000025	0.0000012	0.0026	0.0068	0.00000052	0.093	2.95	0.00022	60.23	0.31	2.28	0.062
OM8109	0.0065	0.0000031	0.0000015	0.0042	0.0069	0.00000041	0.091	3.05	0.00021	58.06	0.099	3.53	0.05
OM8110	0.0068	0.0000032	0.0000012	0.00096	0.0055	0.0000005	0.12	3.24	0.00022	56.55	0.37	1.84	0.044
OM8118	0.0061	0.0000027	0.0000012	0.001	0.0063	0.00000055	0.097	3.13	0.00022	57.87	0.18	2.93	0.32
OM8119	0.0077	0.0000029	0.0000012	0.0035	0.0076	0.00000042	0.08	2.86	0.00022	61.13	0.37	1.59	0.013
OM8123	0.0092	0.000003	0.0000012	0.0043	0.0056	0.00000052	0.11	3.38	0.00022	54.62	0.29	1.72	0.0036
OM8126	0.0084	0.0000029	0.0000012	0.00099	0.0052	0.00000053	0.12	3.5	0.00021	53.41	0.22	1.89	0.001
